# Hyperspectral Imaging for Agricultural and Food Sensing: Applications, Data Analytics, and Deployment Challenges

**DOI:** 10.3390/foods15193396

**Published:** 2026-09-23

**Authors:** Dingbao Wen, Yifan Dong, Lingwei Hu, Dawei Cao, Jitao Li

**Affiliations:** 1 College of Life Science and Agronomy, Zhoukou Normal University, Zhoukou 466001, China; 2212426027@stmail.ujs.edu.cn (D.W.); yifand@zknu.edu.cn (Y.D.); hulingwei2006@126.com (L.H.); 2 School of Physics and Electronic Engineering, Jiangsu University, Zhenjiang 212013, China; 3 School of Physics and Telecommunications Engineering, Zhoukou Normal University, Zhoukou 466001, China

**Keywords:** hyperspectral imaging, crop monitoring, soil assessment, post-harvest quality detection, precision agriculture

## Abstract

Hyperspectral imaging (HSI) combines spatial and spectral information and has been widely investigated for nondestructive sensing in agriculture and food. This review examines HSI applications in crop growth monitoring, abiotic and biotic stress detection, soil assessment, and postharvest quality and safety inspection. Particular attention is given to how acquisition configuration, spectral preprocessing, feature extraction, and machine- or deep-learning models affect performance across laboratory, field, and production-line settings. Recent studies show that red-edge and shortwave-infrared features are useful for estimating chlorophyll, nitrogen, and water status, while learning-based models have been applied to disease detection, contaminant identification, and quality grading. However, transfer across years, sites, and instruments remains difficult, and practical deployment is further limited by calibration inconsistency, hardware cost, and operational complexity. We therefore discuss instrument-aware calibration and preprocessing, model generalization, lower-cost sensing, and deployment strategies that may improve reproducibility and field applicability.

## 1. Introduction

### 1.1. Research Background

Climate change is reshaping the global food security landscape in unprecedented ways, with impacts particularly acute in vulnerable regions such as Sub-Saharan Africa and South Asia. Under high-emission scenarios, average annual temperatures in parts of Sub-Saharan Africa are projected to rise by 2 °C to 6 °C by the end of the century, posing a devastating threat to rainfed agriculture [1], while climate models for South Asia project significant reductions in wheat and maize yields, shortened growing periods, and increased irrigation demands [2,3]. The increasing frequency and intensity of extreme climatic events could expose an additional 20% to 36% of the global population to hunger risk by 2050 relative to moderate scenarios [4], triggering sharp escalations in global food prices when major producing areas suffer from droughts, floods, and heatwaves. This price volatility is further compounded by continued population growth and persistent structural constraints, including poor soil fertility and inadequate irrigation, which leave many regions heavily dependent on erratic rainfall [5]. Although some farmers have been able to partially offset yield losses by expanding irrigation and increasing fertilizer use [6], the compounding pressures collectively drive food insecurity rates far above global averages. Proactive risk management tools such as crop insurance and optimized agronomic practices are therefore being explored in vulnerable countries [7]. The urgency of technological intervention is underscored by projections that, even under moderate scenarios, 453 million people in developing countries will remain undernourished in 2050—well above the SDG target [8].

Effectively addressing these challenges requires timely, large-scale, and accurate information on crop status, soil conditions, and product quality to support adaptive decision-making. Traditional methods—such as visual inspection and laboratory chemical analysis—are often labor-intensive, time-consuming, and destructive [9], and their long detection cycles frequently lead to insufficient timeliness in decision-making [10,11]. In recent years, HSI has emerged as a promising nondestructive technique that can help bridge this gap [12]. By integrating conventional spectroscopy with digital imaging, HSI simultaneously captures two-dimensional spatial information and continuous spectral data across hundreds of narrow wavebands, forming a three-dimensional “hypercube” that enables evaluation of both internal and external quality attributes of agricultural products [13,14]. After standard image preprocessing and spectral extraction, chemometric methods and machine learning models can be applied to quantitatively predict or classify attributes of interest, allowing rapid, non-contact, and multi-constituent analysis [15,16,17,18,19].

This unique capability positions HSI as a versatile tool across the entire agricultural value chain, spanning both pre-harvest and post-harvest sensing. For pre-harvest applications, HSI enables accurate inversion of crop physiological and nutritional traits, detection of abiotic stresses such as drought and salinity, presymptomatic identification of diseases and pests, and comprehensive sensing of soil properties and farmland environments. For post-harvest and food quality, it facilitates nondestructive evaluation of fruit and vegetable quality, assessment of seed quality and vigor, detection of food authenticity and adulteration, and quality inspection of meat, eggs, and processed foods. This review synthesizes recent advances in HSI across these interconnected domains, with the aim of clarifying its current capabilities and limitations, and highlighting pathways toward practical, scalable deployment in support of climate-resilient agriculture and global food security.

### 1.2. Definition and Characteristics of Hyperspectral Imaging

HSI acquires spectral information of a target object in the form of reflectance (passive mode), or in the form of transmittance, fluorescence, or Raman signals (active excitation mode), using an imaging spectrometer with high spectral resolution [20]. HSI systems can be broadly classified according to their acquisition architecture into point-scanning (whiskbroom), line-scanning (pushbroom), wavelength-scanning or staring systems, and snapshot systems. In a staring system, a two-dimensional scene is recorded at one spectral band at a time while the selected wavelength is sequentially tuned, typically using a filter wheel, liquid-crystal tunable filter (LCTF), acousto-optic tunable filter (AOTF), or related tunable element. In contrast, a true snapshot system acquires encoded two-dimensional spatial and spectral information within a single exposure, thereby avoiding inter-band temporal skew. Fourier-transform and computational imaging approaches represent additional spectral-encoding architectures and may overlap with these acquisition categories. These systems decompose the incident light into hundreds of narrow spectral bands (with a spectral resolution typically better than 10 nm, expressed as FWHM), while recording the spatial distribution for each band via a detector array. The final output is a hyperspectral data cube, which integrates two-dimensional spatial information (rows × columns) with one-dimensional spectral information (wavelength), forming a three-dimensional “spatial × spectral” data structure (spatial dimensions x, y; spectral dimension λ). Table 1 summarizes representative acquisition and spectral-encoding architectures for HSI, including their instantaneous sampling manifold, temporal coherence, and signal-to-noise ratio (SNR) properties. Figure 1 illustrates the general data processing workflow for hyperspectral imaging. Similar HSI workflows have been described in previous studies [20,21].

In the field of agriculture, HSI enables non-destructive and rapid monitoring of key parameters such as crop physiological status, pest and disease severity, and soil nutrient content by capturing the spectral reflectance, transmittance, or fluorescence emission characteristics of crop leaves, fruits, or soil. Based on this principle, HSI exhibits the following significant characteristics:

(1) HSI is capable of providing spectral resolution at the nanometer scale, typically with more than 100 bands covering a broad spectral range from the visible to the short-wave infrared. This high resolution enables the precise capture of subtle spectral features (e.g., absorption peaks, reflection valleys) and provides the basis for the identification of complex substances. For example, heavy metal contamination in soil or crop diseases can be diagnosed early using hyperspectral data [26,27].
(2) Image–spectrum integration. HSI not only records the spatial image of the target object but also simultaneously acquires the complete spectral curve for each pixel. This fusion of spatial and spectral information allows the technology to distinguish between objects of similar appearance but different spectral signatures [28]. For instance, in a field environment, HSI can differentiate the spectral signal of crop leaves from the soil background, enabling precise nutrient diagnosis or disease localization [29].
(3) Non-contact, non-destructive monitoring. HSI does not require physical contact or destructive sampling of the target object, and can obtain real-time information about the state of crops or soil in the field [28]. This feature makes it particularly suitable for dynamic monitoring scenarios, such as drones equipped with hyperspectral cameras to quickly scan large areas of farmland, capturing stress responses or nutrient demand changes during crop growth [30].
(4) Multi-dimensional data analysis potential. More importantly, the hyperspectral data cube contains rich information dimensions that can be mined through feature extraction algorithms (e.g., band selection, principal component analysis) or machine learning models (e.g., SVM, deep learning) [26,28]. For example, classification of hyperspectral data via deep learning algorithms can achieve early warning of crop diseases or grading of fruit ripeness [26].
(5) Atmospheric sensitivity and correction. For field, airborne, and spaceborne HSI, atmospheric water vapor, aerosols, and haze may affect the measured signal. When atmospheric path effects are non-negligible, algorithms such as FLAASH, 6S, ATCOR, or QUAC can be applied to retrieve surface reflectance. Atmospheric correction is generally unnecessary for controlled laboratory HSI. For combined thermal stress assessment, HSI reflectance data in the visible and near-infrared can be fused with a separate thermal infrared (TIR) imager (8–14 μm) to monitor canopy temperature variations alongside water status indicators.
(6) Synergy with Complementary Technologies. Beyond its standalone capabilities, HSI’s value is amplified when integrated with other non-destructive sensing modalities. The fusion of HSI with diffraction imaging technologies is used in agricultural diagnostics, where the rich spectral information of HSI can be synergized with the high-resolution microstructural analysis capabilities of diffraction techniques, enabling multiscale characterization of crop health and food quality [31]. Similarly, the combination of HSI with confocal laser scanning microscopy (CLSM) has enabled simultaneous assessment of chemical composition and tissue microstructure in processed meat products [32,33], while integration with gas-chromatography–ion-mobility spectrometry (GC-IMS) provides complementary volatile compound profiles that enhance the interpretation of spectral changes during food processing [33]. These multi-platform approaches offer an understanding of food quality by bridging the gap between molecular composition, microstructure, and sensory attributes. For instance, fusing HSI with an electronic nose has distinguished citrus Huanglongbing from Zn-deficiency with 97.79% accuracy via VOC profiling [34].

### 1.3. Comparison of Hyperspectral Imaging with Traditional Imaging Techniques

Traditional reference methods, including laboratory chemical assays, PCR, and ELISA, can provide high analytical accuracy, but they are often destructive, time-consuming, spatially sparse, or dependent on specialized personnel. Even expert visual inspection can be subjective and experience-dependent [35], and color or textural changes in leafy vegetables often require large-area sampling to capture spatial heterogeneity [36]. HSI should therefore not be regarded as a universally more accurate replacement for conventional methods. Rather, its principal advantages lie in rapid, non-contact, spatially resolved, and potentially multi-attribute screening. The achievable performance of HSI remains dependent on instrument calibration, acquisition conditions, sample variability, and validation design.

In terms of early disease detection, traditional imaging techniques rely on visible symptoms (such as leaf yellowing or lesions), while hyperspectral imaging can capture subclinical changes in crops through reflectance anomalies at specific wavelengths (e.g., the red-edge band at 680–750 nm), enabling early warning of diseases. Regarding precise nutrient diagnosis, multispectral imaging can estimate several key crop traits, including nitrogen, chlorophyll, and water status, using established vegetation indices. However, its limited number of discontinuous bands generally provides less spectral flexibility than HSI for disentangling overlapping biochemical and structural signals—such as separating nitrogen-specific absorption features from canopy architecture or soil background effects. In contrast, hyperspectral imaging, with its hundreds of contiguous narrow bands across the visible and near-infrared to short-wave infrared (VNIR–SWIR) range, can resolve finer spectral features (e.g., protein-related absorptions near 1510 nm and 1940 nm, or cellulose/lignin features in the 2100–2300 nm region), enabling simultaneous quantification of multiple nutrients like nitrogen, phosphorus, and potassium, and supporting simultaneous detection of multiple components. Such multi-parameter capability has been demonstrated for multiple biochemical parameters, including chlorophyll and other quality-related constituents [37]. This capability can support precision fertilization and resource optimization. Furthermore, hyperspectral imaging can distinguish between soil background and crop signals (e.g., water distribution under drought stress), adapting to changing farmland environments. Beyond single-modality analysis, fusing HSI with complementary sensors such as electronic noses can provide a more comprehensive evaluation; for example, combining HSI with an electronic nose has improved pork freshness prediction by correlating spectral–spatial features with volatile organic compound profiles [38]. Moreover, multi-task learning models based on CNN can simultaneously predict multiple quality attributes from a single HSI measurement, further improving analytical efficiency [39]. Table 2 compares the key characteristics of RGB, multispectral, and hyperspectral imaging technologies, including their typical detector and spectral-selection architectures.

### 1.4. Technological Development

The evolution of HSI has been shaped by four principal optical architectures: diffraction grating/prism dispersive systems, tunable-filter systems such as LCTF/AOTF, interferometric/FT-based systems, and snapshot/computational spectral imaging. These architectures impose different trade-offs in spectral resolution, throughput, acquisition speed, spatial–spectral registration, and calibration requirements. Prism and grating dispersers offer high throughput and linear dispersion, respectively, but may suffer from nonlinear dispersion, polarization dependence, or stray light; convex grating Offner and Dyson configurations are widely used in pushbroom imagers for their compactness and image quality. Filter-based tunable elements, including filter wheels, LCTFs, AOTFs, and Fabry–Pérot filters, enable wavelength selection without moving parts or with simple architectures, but trade spectral resolution against tuning speed. FT-based systems acquire an interferogram at each spatial position and retrieve the spectrum via Fourier transform, offering high spectral resolution and multiplex advantage but requiring stable alignment. Computational spectral imaging uses coded apertures or metasurfaces to encode spatial–spectral information, enabling snapshot acquisition with compact form factors, though reconstruction quality depends on the ill-posedness of the inverse problem.

These spectral-encoding strategies are realized through different imaging architectures. Whiskbroom systems scan the scene point by point, offering high SNR and uniform spectral response at the cost of slow acquisition and mechanical complexity. Pushbroom systems capture one spatial line across the full spectral range simultaneously and are the most common architecture for airborne and UAV HSI, but temporal skew between lines and smile/keystone distortions require careful design and post-processing correction. Staring systems capture the full spatial scene at one wavelength band at a time, avoiding spatial scanning but requiring precise inter-band registration and stable illumination. Snapshot systems capture the full spatial–spectral data cube in a single exposure, eliminating temporal skew, but trade off spatial or spectral resolution and require complex reconstruction. Across all architectures, optical aberrations such as smile and keystone are inherent to dispersive designs and must be addressed through wavelength calibration and geometric correction.

In terms of platform evolution, HSI has evolved from laboratory benchtop systems to field-portable devices, UAV-borne pushbroom and snapshot imagers, airborne campaigns, and spaceborne missions. Each platform imposes distinct constraints on weight, power, spectral range, and calibration stability. Early concepts such as NASA’s HIRIS proposal (1987) set the paradigm of acquiring hundreds of contiguous narrow spectral channels to resolve surface “spectral fingerprints,” though it was not flown [44]. The airborne AVIRIS (late 1980s) established the 5–10 nm sampling over 400–2500 nm with exceptional SNR and calibration, becoming the gold standard for algorithm validation [45]. Spaceborne missions surged in the 2010s–2020s, including PRISMA (Italy, 2019), HISUI (Japan, 2019), EnMAP (Germany, 2022), EMIT (NASA, 2022), and China’s ZY1-02D/02E and GF-5.02 [46,47,48,49,50]. These missions have progressively improved radiometric accuracy, spectral resolution, and revisit frequency. Figure 2 summarizes this timeline. The trade-off between spatial resolution, spectral resolution, and swath width remains a central design challenge, and atmospheric correction and BRDF effects continue to pose major challenges for quantitative applications.

### 1.5. A Generalized and Scenario-Dependent HSI Workflow

Before proceeding to a detailed discussion of specific application scenarios, it is necessary to outline the complete technological pipeline from raw hyperspectral data to actionable agronomic decisions, and to establish a concise, traceable methodological framework. Rather than representing a universal processing protocol, Figure 1 provides a generalized analytical framework for HSI. The exact calibration and preprocessing procedures depend strongly on the acquisition environment, optical architecture, detector technology, scanning mode, and illumination configuration. A common analytical backbone can nevertheless be identified, consisting of data acquisition, instrument- and scene-specific calibration, feature representation, model construction and validation, and decision output. The various modelling abbreviations and performance metrics that recur throughout subsequent chapters can all be traced back to this foundational framework.

(1) Data acquisition. The imaging mode (pushbroom being the most common [51]), spectral range (VNIR or full VNIR–SWIR coverage [52]), and carrier platform (laboratory benchtop, field-based handheld/vehicle-mounted, unmanned aerial vehicle, aircraft, or satellite) are selected according to the specific application. The trade-off between spatial resolution and spatial coverage directly defines the envelope of feasible analyses.

(2) Instrument- and scenario-specific calibration and preprocessing. The raw digital numbers (DN) recorded by the sensor are converted into physically meaningful apparent reflectance or radiance. The specific corrections required depend on the instrument architecture and acquisition environment. For laboratory and proximal-sensing systems, dark-current correction, white-reference normalization, flat-field correction, and wavelength calibration are standard. For field/UAV pushbroom systems, additional motion compensation, line registration, geometric correction, and illumination normalization may additionally be required. For airborne and spaceborne systems, atmospheric correction using radiative transfer models such as FLAASH, ATCOR, or 6S is typically required when surface reflectance retrieval is affected by atmospheric path effects, along with orthorectification and BRDF correction [53]. For staring or tunable-filter systems, inter-band registration, bandpass characterization, and illumination stability assessment may be required. Not all corrections are universally required; rather, each acquisition scenario imposes its own minimum calibration and preprocessing requirements. To further improve spectral reproducibility and modelling robustness, Savitzky–Golay smoothing or wavelet denoising is applied to suppress detector noise, while standard normal variate (SNV) and multiplicative scatter correction (MSC) are used to mitigate particle-size effects, surface scattering, and optical path-length variations that cause baseline drift.

(3) Feature engineering and data fusion strategies. This stage prepares optimized inputs for subsequent modelling. It encompasses two complementary sub-tasks. Feature extraction and dimensionality reduction: Because adjacent bands in hyperspectral data are highly correlated, the hundreds of redundant dimensions not only increase computational burden but also risk model overfitting due to the Hughes phenomenon (the “curse of dimensionality”). For unsupervised linear reduction, principal component analysis (PCA) compresses the original spectral space into a few principal components that retain the vast majority of variance information. Supervised wavelength selection methods, on the other hand, directly identify a subset of key wavebands most relevant to the target variable; representative algorithms include competitive adaptive reweighted sampling (CARS), successive projections algorithm (SPA), uninformative variable elimination (UVE), and iteratively retaining informative variables (IRIV) [54]. An alternative strategy, VISSA-SR, combines variable space shrinkage with stepwise regression and has achieved 97.14% accuracy in apple origin classification with reduced modeling time [55]. These approaches not only improve model efficiency but also often yield selected wavelengths that correspond to specific absorption features of chemical bonds, thereby enhancing model interpretability and providing a theoretical basis for designing low-cost, purpose-built multispectral sensors. Multi-modal data fusion: To further break the information bottleneck of a single spectral modality, multi-modal fusion approaches (feature-level, decision-level, and attention-guided cross-modal fusion) integrate complementary heterogeneous data such as thermal infrared temperature, LiDAR point clouds, GC-IMS volatile fingerprints, or confocal microscopy images. Among these approaches, multimodal semantic collaborative classification has been explored by integrating hyperspectral images with LiDAR data, where the two modalities provide complementary spatial and spectral information and improve classification performance [56]. This strategy is particularly crucial for diagnosing complex stresses or quality attributes where spectral signatures alone are insufficient.

(4) Model construction, evaluation, and generalization enhancement. The suitability of different data-analytics approaches depends on sample size, spectral–spatial structure, interpretability requirements, and deployment constraints. Representative data-analytics approaches can be grouped into four broad categories:

Chemometrics (PCA, PLSR, LDA): These methods remain highly effective for small-sample quantitative analysis and offer strong interpretability, as latent variables can often be linked to specific absorption features. Partial least-squares regression (PLSR) extracts latent variables that maximize the covariance between the spectral matrix and the target matrix, establishing linear or weakly nonlinear relationships [57,58].

Conventional machine learning (SVM, RF, XGBoost, KNN): These algorithms capture nonlinear relationships with moderate sample sizes and require less feature engineering than deep learning [59,60]. They are widely employed for disease classification, stress-level discrimination, and adulteration detection.

Deep learning (1D/2D/3D CNNs, CNN-RNN, Transformers): These methods excel when large labeled datasets are available and when joint spatial–spectral feature learning is beneficial [61,62]. 1D-CNNs are well suited for pixel-wise spectral classification, 2D/3D-CNNs exploit spatial–spectral joint representations, and Transformers capture long-range dependencies across spectral bands. However, deep learning is not universally superior: with small sample sizes, deep models are prone to overfitting, and their performance may degrade below that of PLSR or SVM. Autoencoders are also used for unsupervised feature learning and anomaly detection.

Emerging learning paradigms (self-supervised learning, MAE, domain adaptation, transfer learning): Autoencoders and self-supervised learning methods such as masked autoencoders (MAE) are gaining attention for their ability to learn general-purpose representations from unlabeled spectral data, reducing annotation dependence and improving few-shot generalization. Transfer learning and domain adaptation address the performance degradation that occurs when models are transferred across different sensors, years, or ecological zones. One-class classification (OCC) methods require training only on authentic samples, circumventing the impracticality of enumerating all possible adulterant types. Table 3 summarizes these four categories, including representative methods, key strengths, and suitable conditions.

Model selection and hyperparameter tuning should be performed using only the training data, for example through k-fold or leave-one-out cross-validation, followed by evaluation on an independent test set. A critical but often underreported issue in HSI-based classification studies is the risk of data leakage arising from improper sample partitioning. Many studies report remarkably high accuracies (occasionally approaching 100%) using pixel-level random splitting, where individual pixels from the same physical sample—such as a single fruit, seed, or leaf—are distributed across both training and test sets. Because pixels from the same object are spatially and spectrally correlated due to shared biochemical composition and surface properties, this practice artificially inflates performance metrics and yields optimistic results that do not generalize to independent samples. To ensure reliable model evaluation, the following reporting standards are strongly recommended: (1) sample-level partitioning, where all pixels from the same biological or physical unit are assigned exclusively to either training, validation, or test sets; (2) clear specification of dataset sizes, including the number of independent samples (not pixels) in each partition; (3) class balance information, ideally with confusion matrices or per-class precision/recall; and (4) explicit description of the data split method (e.g., random stratified split, leave-one-sample-out cross-validation, or independent external validation with temporally or spatially separated data). Without such transparency, reported accuracies—especially those exceeding 95%—should be interpreted with caution, as they may reflect overfitting to pixel-level correlations rather than true model robustness. Journals and peer reviewers are encouraged to mandate these reporting practices to enhance the reproducibility and practical relevance of HSI research.

(5) Deployment and decision output. The trained model is deployed onto laboratory workstations, in-line sorting systems on production lines, real-time analysis modules on UAVs, or cloud-based decision platforms, generating outputs such as compositional concentration distribution maps, disease risk heatmaps, quality grading results, or agronomic prescription maps [63,64]. However, the transition from laboratory validation to large-scale deployment faces significant hurdles. These include the environmental adaptability of sensors, cross-year/region model robustness, and the interpretability and actionability of outputs for end-users—challenges that will be systematically examined in Section 4.

The final performance of the models is quantified by a unified set of evaluation metrics. For regression tasks, the coefficient of determination (R^2^) and root mean square error (RMSE) measure goodness-of-fit and prediction error magnitude, respectively; RPD is often reported as a supplementary indicator of predictive performance, although its interpretation is application-dependent and should not be treated as a universal threshold [65]. For classification tasks, accuracy, F1-score (the harmonic mean of precision and recall), and the area under the receiver operating characteristic curve (AUC) are used to comprehensively assess discriminative performance.

### 1.6. Objectives of the Review

Existing reviews have provided detailed summaries of HSI applications in individual crops, food products, and specific data-analysis methods. Fewer studies, however, have considered pre-harvest and postharvest applications within the same discussion while also examining how instrumentation, calibration, model transfer, and deployment conditions affect reported performance.

Accordingly, this review organizes recent HSI studies across crop and soil sensing and postharvest food-quality assessment, with particular attention to the links among acquisition configuration, data processing, model validation, and practical deployment. It also discusses recurring limitations related to cross-instrument data compatibility, transfer across seasons and locations, hardware cost, and operational complexity. The aim is to provide a broad but technically grounded overview rather than to propose a single universal workflow or deployment pathway.

## 2. Agricultural Application Scenarios of Hyperspectral Imaging

This chapter focuses on non-destructive sensing of crop status during the field growth period, addressing two core questions: the stresses experienced by the crop and the suitability of the soil environment. Hyperspectral imaging, with its high spectral resolution, integrated spatial–spectral information, and non-destructive capability, provides a technical means to answer these questions. Centered on key scenarios—crop physiological and nutritional diagnostics, abiotic and biotic stress surveillance, and soil environmental quality assessment—this chapter presents the practical application of HSI, and evaluates its technical advantages and current limitations through representative case studies. Figure 3 summarizes the major hyperspectral imaging application scenarios in agriculture.

### 2.1. Inversion of Physiological Parameters

The physiological and nutritional status of crops constitutes the biological foundation for yield and quality formation. Among the numerous potential indicators, chlorophyll content determines photosynthetic capacity and reflects nitrogen nutritional status; nitrogen is the core element for protein synthesis and the primary nutrient factor limiting crop yield; and water serves as the essential medium for cell turgor and metabolic activity. Together, these three parameters form the core indicator system for crop growth monitoring. The capability of HSI to sense these three types of indicators has been extensively validated, and certain applications have reached a considerable level of maturity.

Estimation of chlorophyll content: In terms of crop growth monitoring, hyperspectral imaging can accurately estimate chlorophyll content [66]. As the core pigment of crop photosynthesis, chlorophyll content is a key indicator of plant health status. The retrieval of chlorophyll relies on its characteristic spectral features in the visible to near-infrared region: (a) a well-defined absorption minimum centered at 670–680 nm (chlorophyll a/b absorption) [67]; (b) a steep reflectance increase between ~680 nm and ~750 nm, known as the red-edge region, where the inflection point (red-edge inflection point, REIP) derived from the first derivative spectrum shifts toward longer wavelengths with increasing chlorophyll content [67]; and (c) the slope and area of the red-edge shoulder, which are sensitive to chlorophyll concentration. Beyond single-band or derivative-based features, a suite of well-established vegetation indices has been developed for chlorophyll estimation, including the Modified Chlorophyll Absorption Ratio Index (MCARI), the Chlorophyll Index Red-Edge (CIred-edge), the TCARI/OSAVI ratio (which minimizes soil background effects), and the Red-Edge Normalized Difference Vegetation Index (NDVI705). These indices combine reflectance at strategically selected bands (e.g., 705 nm, 750 nm) to mitigate confounding factors such as leaf structure and canopy geometry [68,69]. For example, in wheat growth monitoring, chlorophyll content can be quantitatively assessed using red-edge-based indices, which can be used for precision fertilization [66,68]. Table 4 summarizes representative red-edge vegetation indices for chlorophyll estimation, highlighting their primary applications, reliance on red-edge bands, and key spectral characteristics.

Beyond traditional vegetation indices, machine learning and deep learning algorithms have been extensively employed to improve the accuracy and robustness of physiological parameter inversion. For example, in maize, deep convolutional neural network (DCNN) models have been successfully applied to classify seed varieties and predict oil content based on hyperspectral data, achieving superior performance compared to conventional methods [73]. Similarly, for a wider range of grains, HSI combined with advanced chemometric methods has enabled the non-destructive determination of key quality attributes, including moisture, protein, and starch content. Furthermore, deep learning approaches such as stacked sparse autoencoders (SSAE) have been used to extract deep spectral features for accurate identification of maize seed varieties [74].

A representative maize study further demonstrated that chlorophyll-related variations are concentrated around the red-edge region and vary systematically with canopy leaf position [75]. Beyond chlorophyll quantification, the spatial distribution of chlorophyll density has been exploited to simultaneously diagnose nitrogen, magnesium, and potassium deficiencies in cucumber leaves, with distinct spatial patterns—uniform chlorosis for N, interveinal for Mg, and marginal for K—achieving 96.67% diagnostic accuracy [76].

Mapping of Nitrogen Content: Hyperspectral imaging combined with machine learning enables simultaneous, non-destructive retrieval of leaf nitrogen content (LNC) and mapping of soil nitrogen, supporting variable rate fertilization (VRF) in precision agriculture. This capability arises from the high sensitivity of vegetation reflectance in the VNIR–SWIR region to nitrogen status, which manifests in several diagnostic spectral features. In the visible range, the green peak (~550 nm) varies with chlorophyll content, which is nitrogen-dependent, so nitrogen stress raises green reflectance; the red absorption trough (670–680 nm) weakens with decreasing nitrogen, while the red-edge region (700–750 nm) shows a “red shift” with higher chlorophyll and a “blue shift” under deficiency—confirmed in maize [77] and wheat [78], where red-edge parameters outperform traditional indices for LNC estimation. Near-infrared (800–950 nm) reflectance, mainly driven by leaf structure and biomass, serves as an auxiliary predictor. In the SWIR region, the 1510 nm band (associated with protein absorption) is sensitive to LNC, typically showing a negative correlation in raw reflectance; together with the 1940 nm water-sensitive band, it helps correct for moisture interference. Both bands have been effectively used in tomato [67] and grassland studies [79]. Machine learning models integrate these multi-band signals to overcome the saturation issues typical of single vegetation indices, with algorithms such as SVM, RF, CNN, and Vision Transformer achieving high accuracy. For instance, a deep learning model utilizing red-edge, 1510, and 1940 nm features attained an R^2^ = 0.92 for rice [80], and even conventional multiple linear regression (MLR) has been applied for soybean nitrogen mapping using red-edge and SWIR data [81]. The key information (crop types, spectral bands, and modeling methods) of these representative studies is summarized in Table 5. By capturing nitrogen-related spectral responses across the VNIR–SWIR range and harnessing the fitting power of machine learning, hyperspectral imaging enables precise, multi-scale nitrogen diagnosis, supporting real-time VRF decisions, improving nitrogen use efficiency, reducing environmental pollution, and promoting sustainable agriculture. Beyond direct nitrogen mapping, this spectral–modeling framework has been successfully extended to early yield prediction: multi-temporal UAV imagery combined with a CNN model predicted rice yield approximately one month in advance in breeding trials, achieving an R^2^ = 0.73 and substantially outperforming conventional methods [82], which further highlights the agronomic value of nitrogen-sensitive spectral information. End-to-end CNN architectures have also been applied to horticultural crops, directly predicting soluble solids content (SSC) and pH in lettuce from raw hyperspectral data without manual feature engineering [83].

Monitoring of water stress: Water status directly influences cell expansion, stomatal conductance, and photosynthetic metabolism, and water deficit remains a primary limiting factor in rain-fed agriculture worldwide. In the near-infrared (NIR) and SWIR regions, water exhibits several characteristic absorption bands: 970 nm (O–H second overtone), 1200 nm (O–H first overtone), 1450 nm (O–H first overtone), and a strong absorption feature at 1940 nm (O–H combination band). As leaf water content decreases, reflectance at these bands increases due to attenuated absorption. The Water Index (WI) and Normalized Difference Water Index (NDWI) have been shown to sensitively capture such variations. At the canopy scale, the Crop Water Stress Index (CWSI)—which exploits the relationship between canopy–air temperature difference and transpiration rate—necessitates thermal infrared (TIR) data, representing a typical scenario of multimodal fusion: HSI supplies biochemical indicators, while TIR provides physical evapotranspiration signals. Water stress directly suppresses dry matter production, leading to alterations in canopy coverage and structure. In turn, the high sensitivity of NIR reflectance to leaf area index (LAI) and canopy architecture, together with the capacity of cellulose and lignin absorption features in the SWIR region (2100–2300 nm) to indicate dry matter, allows biomass to serve as both an auxiliary variable for diagnosing water stress and an integrated proxy for long-term water status. Empirical models based on vegetation indices generally perform satisfactorily prior to canopy closure but tend to saturate thereafter. MSLPP feature extraction—preserving both global variance and local structure—combined with ESMA-optimized SVR achieves R^2^ = 0.9755 for rice moisture detection without reliance on vegetation indices [84]. Accurate evapotranspiration modeling, a key component of water stress diagnosis, relies on proper parameterization of canopy and aerodynamic resistances, which have been systematically investigated for greenhouse cucumber and paddy rice using Penman–Monteith and Bulk Transfer approaches [85,86,87]. Incorporating canopy height data derived from LiDAR or photogrammetric structure-from-motion has been demonstrated to effectively overcome this saturation bottleneck. In sparse canopies, however, SWIR-based water and biomass signals are subject to considerable interference from soil background and non-photosynthetic vegetation components. The fusion of HSI with TIR, LiDAR, and chlorophyll fluorescence data is increasingly used for simultaneously disentangling the coupled effects of water status, nitrogen nutrition, and canopy structure. Furthermore, soil amendments such as biochar can improve soil physical properties and water-holding capacity; the resulting shifts in soil spectral background may interact with crop water signals. This underscores the need for future water-stress diagnostic models to incorporate soil management history (e.g., biochar application) as a covariate, thereby ensuring robust and context-aware stress diagnosis [88].

### 2.2. Stress Response Analysis

Abiotic stresses—salinity, heat, and heavy metals—are key environmental constraints on crop productivity. Unlike biotic stresses, the spectral responses to abiotic stress are typically systemic and progressive, involving multidimensional changes in leaf pigments, water status, and cellular structure.

Salinity stress: High soil salinity lowers the osmotic potential of the rhizosphere, making water uptake difficult and triggering physiological drought and ion toxicity. The characteristic spectral responses include a general increase in SWIR reflectance as cellular dehydration alters light scattering pathways, elevated visible reflectance due to chlorophyll degradation, and a blue shift of the red edge. Salt stress indices (SSI) are typically constructed as ratios of salt-sensitive to reference bands; however, because of differences in salt-tolerance mechanisms among crops, the optimal SSI band combination requires crop-specific calibration. A noteworthy phenomenon is the spectral dynamics of the re-watering recovery effect—after stress is relieved, changes in SWIR reflectance precede the recovery of visible-range chlorophyll indicators, suggesting that SWIR can serve as a sensitive probe for early stress alleviation. This has practical implications for water-saving irrigation scheduling. Studies on oilseed rape (*Brassica napus* L.) have shown that re-watering after moderate salt stress can restore net photosynthetic rate by 44.9–63.0%, with re-watering water use efficiency reaching its optimum under moderate stress [89], providing a physiological basis for SWIR-based detection.

Heat stress: High temperature directly damages the oxygen-evolving complex of photosystem II (PSII) and the thylakoid membrane structure, reducing photosynthetic electron transport efficiency and increasing excess excitation energy. The most robust spectral fingerprint is a blue shift of the red edge: chlorophyll degradation and damage to the photosynthetic apparatus shift the red-edge position by 1–5 nm toward shorter wavelengths, accompanied by decreases in red-edge slope and area. These changes usually occur before leaf yellowing or curling becomes visible, giving HSI early warning capability. Sun-induced chlorophyll fluorescence (SIF) is the signal re-emitted from PSII when absorbed light energy is not used by photochemistry or dissipated as heat; it can be retrieved from hyperspectral data using the depth changes of Fraunhofer lines (e.g., the O2-A band at 760 nm). SIF responds to heat stress even more rapidly than reflectance—while PSII function is impaired but chlorophyll has not yet significantly degraded, SIF has already begun to decline. Therefore, SIF and red-edge blue shift constitute complementary early-to-mid stage heat stress indicators. Further fusion with thermal infrared (TIR)-derived canopy temperature anomalies forms a three-modal (spectral + fluorescence + thermal) early warning system, reducing the false alarm rate of single indicators. For instance, greenhouse studies on tomato demonstrated that micro-spray cooling reduced leaf temperature by 3–6 °C and improved the effective quantum yield of PSII (ΦPSII) [90]; a combined micro-spray and drip irrigation (MSDI) system lowered leaf temperature by 4.9 °C and increased ΦPSII by 40–53% in cucumber and 49–62% in tomato, along with improvements in net photosynthetic rate and stomatal conductance [91]. Studies on rice canopies have confirmed that red-edge parameters are significantly correlated with SPAD values and leaf area index (LAI), effectively quantifying cumulative heat stress [92], while SIF has demonstrated high sensitivity to heat-induced photosynthetic depression before structural changes become apparent [93]. In vineyard applications, multi-variable models that integrate red-edge spectral features with thermal infrared imagery further improve early warning capability for heat stress [94].

Heavy metal stress: Heavy metals such as cadmium, lead, and copper are taken up by roots and subsequently disrupt enzyme systems, inhibit chlorophyll synthesis, and induce oxidative stress. Their spectral detection faces a fundamental challenge: HSI cannot directly “see” metal atoms within plant tissues but can only sense the resulting physiological and biochemical consequences. Consequently, the spectral response to heavy metal stress is indirect and overlaps with those of drought and nutrient deficiency—severely limiting specificity under field conditions with mixed stresses. Reported sensitive bands include ~480 nm (associated with changes in carotenoids and flavonoid secondary metabolites), the red-edge region (chlorophyll damage), and 1780 nm and 2200 nm (related to alterations in cell wall constituents and nitrogenous compounds). Algorithms such as wavelet support vector regression (WSVR) and bootstrapping soft shrinkage PLSR (BOSS-PLSR) have achieved good prediction accuracy in controlled hydroponic experiments. For instance, WSVR combined with Vis-NIR HSI predicted cadmium (Cd) content in lettuce leaves with R_p_^2^ of 0.8843 and RMSEP of 0.1292 mg/kg, enabling visualization of within-leaf Cd distribution heterogeneity [95]. Similarly, BOSS-PLSR using only five optimal wavelengths (552.5, 556.7, 572.0, 701.8, and 711.3 nm) achieved R_p_^2^ of 0.9821 for Cd in tomato leaves [96]. However, moving from “correlation” to “causation” remains the core challenge. The future breakthrough lies in coupling radiative transfer models (e.g., the PROSPECT leaf optical model) with plant heavy metal toxicity mechanisms (oxidative damage, osmotic adjustment substance accumulation) to construct a hybrid inversion framework with physical and physiological constraints. This would allow spectral retrieval to go beyond statistical association and trace back to mechanistically interpretable intermediate variables.

### 2.3. Early Warning of Diseases and Pests

Biotic stresses, primarily diseases and insect pests, are major factors causing crop yield loss and quality deterioration. The value of HSI lies not only in post-symptomatic diagnosis but, more critically, in early warning during latent or asymptomatic stages, thereby buying time for precision pesticide application and area-wide control.

Disease Detection: Although diseases and pest infestations exhibit distinct macroscopic symptoms, many biotic stresses share two highly conserved spectral response pathways. The first is the disruption of mesophyll cell structure: invasion by pathogens or pests damages the cell–air interfaces within leaves, weakening multiple scattering in the near infrared (NIR, 750–1300 nm) and thereby markedly reducing reflectance. The second is the induction of chlorophyll degradation: this causes a blue shift of the red edge, diminished green-peak/red-trough contrast, and the accumulation of secondary metabolites (phenolics, flavonoids, anthocyanins) that shift the visible hue away from green. These two pathways may be asynchronous—tissue damage can lag behind pigment changes—but together shape the characteristic spectral signatures of disease.

Fusarium head blight (FHB), caused by *Fusarium graminearum*, not only reduces grain yield but also produces deoxynivalenol (DON), threatening food safety [97]. At the canopy scale, FHB detection exploits the reduced reflectance of infected wheat spikes in the visible–NIR region and abnormal absorption features in the SWIR arising from fungal mycelia and toxin metabolites. By calculating spectral indices such as the Wheat Fusarium Spectral Index (WFSI) and the Red-Edge Head Blight Index (REHBI)—the latter specifically designed for Sentinel-2 multispectral imagery [98]—and combining them with SVM classifiers, FHB can be detected prior to visible symptoms, achieving 85–96% spike-level accuracy before harvest [98,99]. This provides a direct basis for zonal harvesting and mycotoxin risk early warning. Notably, the spectral manifestation of Fusarium diseases is closely tied to the diversity and toxigenic profiles of the pathogen; a systematic survey of rice Fusarium communities revealed that different species complexes (e.g., FIESC, FFSC, FSAMSC) exhibit distinct toxigenic and pathogenic characteristics [100], offering a biological basis for spectrally discriminating fungal stresses. Near-infrared hyperspectral imaging (NIR-HSI) combined with partial least squares regression for direct quantification of DON in single wheat kernels has been validated by Antoni Femenias et al., with optimal PLSR models achieving RPD values above 2.0 [101]. The prevalence and toxigenic diversity of Fusarium in rice in eastern China were demonstrated by Chen et al. (2023), who detected DON contamination in newly harvested rice from Jiangsu and confirmed its origin from field-occurring Fusarium head blight, indicating that Fusarium can infect rice and produce type-B trichothecenes including DON [102].

Citrus Huanglongbing (HLB) is the most devastating citrus disease globally. HLB is unique in that the phloem-limited pathogen *Candidatus Liberibacter asiaticus* triggers substantial starch accumulation in leaves [103], producing SWIR spectral responses (e.g., at 1450, 1940, and 1200 nm, bands associated with starch–water interactions) that are distinct from common nutrient deficiencies [103,104]. In the visible spectrum, HLB causes chlorophyll degradation and a characteristic blue shift of the red-edge position (approximately 710–715 nm), which serves as a sensitive indicator of early infection [105]. Deep architectures such as ResNet have shown preliminary promise in detecting asymptomatic HLB samples across several independent datasets, but their reliability in generalizing to different cultivars and regions still requires large-scale, multi-center validation [106]. A complementary approach based on starch-induced optical property changes—polarized light transmission imaging combined with Stokes vector analysis—has achieved 96.67% classification accuracy for nondestructive detection of abnormal starch accumulation in HLB-infected leaves [107]. To address the complex diagnostic demands of multiple co-occurring diseases in the field, multi-model fusion networks (MMFN) integrate outputs from several heterogeneous base models (e.g., sub-models specialized in spectral features, spatial textures, and disease-specific prior knowledge). An MMFN combining AlexNet, VGG, and ResNet achieved >98% accuracy for multiple citrus diseases, and a further ensemble strategy incorporating transfer learning attained an average accuracy of 98.68% across multiple citrus leaf disease categories [108]. These advances improve classification robustness over single models and represent a paradigm shift from “one-to-one” to “one-to-many” in spectral disease diagnosis.

Insect pest monitoring: Damage from piercing-sucking pests (aphids, planthoppers, leafhoppers, etc.) exhibits a characteristic temporal spectral progression. In the early stage, stylet insertion causes local cell content loss, but overall canopy spectral changes remain subtle. In the mid-stage, sustained feeding induces water deficit—leading to increased reflectance in water-absorption bands near 970 and 1450 nm—and honeydew secretion, with subsequent sooty mold growth darkening the visible reflectance. In the late stage, systemic chlorophyll decline, leaf curling, and premature senescence produce a pronounced blue shift of the red edge and a marked decrease in NIR reflectance.

Taking aphids as an example, feeding lowers chlorophyll a and b concentrations, which raises reflectance at the green peak (~550 nm) and weakens absorption at the red trough (~680 nm) in the visible domain. In the NIR region (750–1300 nm), damage to mesophyll cell architecture reduces internal multiple scattering, causing a marked reflectance decrease; under aphid stress, cotton leaves show a positive correlation between NIR reflectance (748–863 nm) and chlorophyll content [109]. In the SWIR domain, water deficit alters the depth of absorption features near 1450 and 1900 nm [110]. Quantitative estimation of wheat aphid density at the leaf level was achieved using continuous wavelet analysis and PLSR, yielding an R^2^ of 0.77 [111]. Furthermore, Fisher linear discriminant analysis (FLDA) based on the Greenness Index (GI) and the first-derivative parameter Dy can effectively discriminate aphid-infested leaves from those affected by powdery mildew or yellow rust, with classification accuracies exceeding 90% in wheat and mustard [112].

This well-defined temporal evolution enables multi-temporal HSI monitoring. By acquiring high-frequency UAV spectral data at critical growth stages, time-series models such as LSTM can learn the spectral trajectory and issue early warnings 3–5 days before pest outbreaks. For instance, an LSTM-Attention model achieved optimal performance in 7-day time-series prediction of tea whiteflies, with an RMSE of 2.84 [113]; however, for rice brown planthopper, since stem-base damage precedes visible canopy symptoms and produces no significant NDVI anomaly, top-down UAV imagery alone cannot yet achieve early identification [114]. Leaf-level hyperspectral characterization of coffee leaf miner identified the red-edge region (~700 nm) as the most effective for discriminating infestation severity [115].

For rice planthoppers, the brown planthopper (*Nilaparvata lugens*) disrupts the canopy structure by accelerating chlorophyll degradation and altering leaf morphology, resulting in reflectance anomalies in the red-edge band (centered at approximately 710 nm). Integrating UAV-borne HSI with time-series analysis and machine learning models (e.g., gradient boosting machines and recurrent neural networks) captures the pest-driven spectral evolution, enabling field-scale prediction of planthopper outbreak probability over a 5–7 day forecast horizon [116]. A further critical contribution of insect pest monitoring is driving the paradigm shift toward “on-demand” pesticide application. Conventional uniform spraying is replaced by variable-rate application guided by hyperspectral pest distribution maps, where fields are zoned by infestation severity, reducing pesticide use by 30–50% [116]. Enhanced spectral resolution is key: the spectral differences among healthy plants, early-infested plants, and bare soil in narrow bands far exceed those captured by broadband vegetation indices, providing the physical basis for high-precision pest distribution mapping. The principles of spectral response mechanisms and machine learning algorithms for plant disease detection have been systematically reviewed [117].

### 2.4. Environmental Monitoring of Soil and Farmland

Soil is the growth substrate for crops, and its physicochemical properties and contamination status directly influence root development, nutrient uptake, and agricultural product safety. The application of HSI in soil sensing spans multiple dimensions, from fertility indicators to emerging pollutants.

Soil organic matter (SOM). The OSC algorithm effectively eliminates environmental interferences such as soil moisture from satellite hyperspectral imagery, significantly improving texture fraction estimation accuracy, with CARS-selected characteristic bands concentrated at 500, 750, 900, and 2200 nm [118]. SOM is a core indicator of soil fertility, influencing aggregate structure, water and nutrient retention capacity, and microbial activity. It exhibits significant spectral absorption characteristics in the SWIR region at 2200–2500 nm, primarily originating from the second harmonic and combination bands of C-H (aliphatic and aromatic), N-H (amine and amide), and C-O (carboxyl) functional groups commonly present in soil organic compounds, with absorptions near 2300–2350 nm attributed to C-H combination bands and those in the 2100–2200 nm and 2400–2500 nm regions involving contributions from N-H and C-O groups [119]. Based on PLSR models—a multivariate statistical method capable of effectively handling the high dimensionality and strong collinearity inherent in hyperspectral data—HSI can establish robust mappings between spectral reflectance and SOM content, enabling rapid estimation [119]. Preprocessing techniques such as standard normal variate (SNV) and multiplicative scatter correction (MSC) are essential for eliminating scattering effects caused by soil particle size, moisture, and surface roughness, which are key interfering factors affecting estimation accuracy. SOM retrieval achieves excellent accuracy on laboratory-dried and ground soil samples, but under in-situ field conditions, accuracy typically drops due to moisture interference and surface heterogeneity. In large-scale rapid surveys, HSI offers a practical alternative to conventional laboratory analysis, achieving good consistency with chemical results while significantly improving throughput and reducing cost. Combined with characteristic wavelength selection and machine learning optimization strategies, this non-destructive, high-efficiency approach provides a scientific basis for dynamic monitoring of soil fertility and carbon stock assessment across regional scales. Beyond stock assessment, precipitation regimes have been shown to significantly modulate ecosystem respiration (RE) in agricultural regions, with implications for regional carbon–climate feedbacks [120]. Some researchers have proposed a method for estimating soil transfer function based on hyperspectral remote sensing images, which can be used to obtain soil spectral reflection, as shown in Figure 4 [118].

Soil microplastic (MP) detection: Soil microplastic pollution has emerged as a significant environmental concern in recent years. Common plastic polymers such as polyethylene (PE), polypropylene (PP), and polystyrene (PS) exhibit characteristic reflectance features in the near-infrared (NIR) and SWIR regions, primarily associated with C–H stretch overtones and combination bands of polymer chains, with different polymers displaying distinguishable absorption peak patterns in the 1000–2500 nm range [121]. Studies have demonstrated the feasibility of using HSI combined with machine learning classifiers such as SVM and random forest (RF) to detect and visualize microplastics on soil surfaces under laboratory conditions, and the SWIR region provides richer polymer chemical information compared to VNIR, facilitating improved discrimination [122]. However, current HSI techniques still face a sensitivity bottleneck for reliable quantification of low concentrations of microplastics in real soil environments (typically at mg/kg levels, well below 0.5%), as soil matrix components including organic matter, clay minerals, moisture, and iron oxides severely mask the weak signals of polymers. By constructing targeted spectral indices (e.g., the Band Difference Index, BDI) and combining them with advanced classification models (e.g., RF, convolutional neural networks, and Transformer architectures), HSI can provide spatial distribution information on microplastic-contaminated areas, supporting pollution source identification and risk assessment [123]. These limitations may be addressed through spectral signal enhancement, multi-modal data fusion, and standardized spectral databases.

Soil salinity is another major degradation driver in coastal agricultural regions. Field surveys combined with remote sensing data have revealed that seawater intrusion and climatic shifts led to an 8.6% vegetation decline and a 4.4% expansion of barren land over nearly three decades in Pakistan’s Sujawal district, with over 65% of topsoil samples exhibiting elevated electrical conductivity [124]. In terms of methodology, recent studies have further enhanced salinity inversion accuracy by integrating multi-source remote sensing (RGB, multispectral, thermal) with stacking ensemble learning, achieving R^2^ improvements of 6–9% over single models in barley-cultivated salinized soils [125]. Beyond data fusion, further studies have shown that crop-type classification and multi-temporal analysis also contribute to salinity estimation accuracy, with artificial neural networks achieving an R^2^ of 0.72 for soil salt content prediction using Sentinel-2 imagery in sunflower and maize fields [126]. The broader trajectory of saline soil remediation research has shifted from physical-chemical interventions toward biological and integrated strategies [127].

Soil compaction diagnosis: Soil compaction leads to increased bulk density and reduced porosity, impeding root penetration and water infiltration. Due to the decrease in porosity, the reflectivity of compacted soil in the short-wave infrared band (1900–2100 nm) is significantly reduced [128]; this band is highly sensitive to overtone and combination absorptions of water molecules, and compaction leads to tighter particle arrangement, reduced multiple scattering paths, and decreased water retention capacity in micropores, thereby weakening water absorption features and lowering overall reflectance. Micro-CT revealed that compaction shifts pores toward smaller radii, increasing water retention but reducing hydraulic conductivity [129]; vision-based tracking of plate markers showed strong correlation between amplitude and dynamic modulus (R^2^ > 0.85), enabling real-time assessment [130]. Based on spectral texture features (e.g., contrast, correlation, energy, and homogeneity extracted from the Gray Level Co-occurrence Matrix, GLCM) combined with RF models, HSI can effectively evaluate the degree of soil compaction—with fusion of spectral and texture features achieving prediction coefficients of determination (R^2^) exceeding 0.85 for soil bulk density, significantly outperforming single-spectral models [131]. This capability provides spatially explicit decision support for “on-demand subsoiling” operations, avoiding energy waste and secondary soil structure damage from over-tillage, and can be further integrated with Digital Soil Mapping (DSM) frameworks to construct dynamically updated soil compaction risk maps, offering important application value in farmland environmental monitoring and management.

## 3. Post-Harvest and Food Quality Sensing

This chapter focuses on the non-destructive detection of quality, safety, and authenticity of agricultural products throughout the entire chain from harvest to the consumer end. The integration of hyperspectral imaging with deep learning now dominates post-harvest quality research, with applications spanning safety inspection, external defect detection, mechanical damage assessment, maturity evaluation, nutrient quantification, and firmness measurement [132]. The core distinctions from the field crops discussed in Section 2 lie in the following aspects: the detection objects shift from living plants to detached post-harvest products; the spectral responses transform from dynamic reflections of physiological processes to static characterizations of chemical composition and physical structure; and the detection tasks evolve from monitoring what the crop is undergoing to evaluating whether the product is acceptable in quality, genuine in authenticity, and safe for consumption. Xing and Chen [133] systematically summarized recent advances in HSI for quality and safety detection across fruits, vegetables, meat, grains, and tea, covering nutrients, moisture, variety/origin identification, adulterants/additives, heavy metals, and pesticide residues. This chapter covers a broad range of product types, from fresh fruits and vegetables to processed foods.

### 3.1. Fruit Quality Grading

The eating quality of fruits and vegetables hinges on consumer experience (sweetness, firmness, flavor) and shelf life (storability, disease resistance). HSI has demonstrated strong industrial potential for the detection of SSC and early-stage mold contamination.

SSC and firmness: SSC, consisting primarily of soluble sugars, and firmness are core indicators of fruit maturity and eating quality. The O-H and C-H bonds in sugar molecules give rise to overtone and combination absorptions in the near-infrared (NIR) region (780–2500 nm), with bands near 960, 1180, 1450, and 1940 nm being particularly sensitive to sugar concentration. PLSR is the most widely used baseline method for SSC prediction, compressing the full spectrum into a small number of latent variables to build a linear mapping to SSC. Deep learning approaches have further improved accuracy: multi-attention convolutional neural networks (MA-CNN) that extract spectral and spatial texture features in parallel and fuse them via attention weighting have achieved an R^2^ of 0.960 for SSC prediction in Fuji apples, outperforming conventional PLSR [134].

Cross-year model maintenance is a practical challenge for SSC prediction. Spectral responses drift from year to year due to differences in light, temperature, and rainfall, causing models trained on a single season to degrade in subsequent years. Slope/Bias correction—using a small set of new-season samples to regress predicted values against measured values and applying the resulting linear correction to the original model outputs—provides a lightweight model updating strategy. A long-term study on Fuji apples demonstrated that a PLSR model calibrated with samples from 2012–2013 could maintain good prediction performance (RMSEP of 0.500–0.637%) over five successive years when combined with Slope/Bias correction and effective wavelength selection (CARS-SPA) [135], avoiding the need for full retraining. Beyond temporal robustness, portability is another key to moving HSI from the laboratory to the orchard and the grading line. Wavelength selection algorithms can compress the full spectrum to a small set of critical bands, enabling low-cost narrowband filter-based devices to replace expensive spectrometers. For apple SSC, UVE-SPA-MLR selected only 21 wavelengths and achieved R_p_ = 0.951 [136]. In studies on intact apples, even when employing advanced portable near-infrared (NIR) spectrometers and optimized chemometric calibration models, the root mean square error of prediction (RMSEP) typically falls within the 0.5–0.8 Brix range [137]. For grape SSC, a novel VMD-RC (Variational Mode Decomposition–Regression Coefficients) wavelength selection method outperformed conventional CARS and IRIV algorithms, achieving R_p_^2^ = 0.93 and RMSEP = 0.6% using only 12 optimal wavelengths [138]. Deep learning models such as stacked autoencoders with size compensation further improved grape TSS and TA prediction to R_p_^2^ > 0.92 [139]. These portable HSI/NIR devices are now entering trial commercial use for postharvest sorting of kiwifruit, mango, cherry, and other fruits. The same approach has been extended to powdered foods, enabling simultaneous prediction of caffeine, polyphenols, amino acids, and chlorophyll in matcha using CARS- and BOSS-PLSR models [140].

Mold and fungal contamination: Postharvest mold is a major cause of fruit and vegetable loss. Fungal mycelia and metabolites (e.g., organic acids, mycotoxins) exhibit characteristic absorption bands at 1200 nm (C-H), 1450 nm, and 1940 nm (O-H). These spectral changes occur 24–48 h before visible fungal colonies appear, giving HSI an early detection capability. Convolutional neural networks (CNNs) leverage both spectral information and the spatial texture of mold patches (the radial diffusion pattern of mycelia) to achieve high-accuracy early mold segmentation in strawberries, apples, and citrus, outperforming conventional algorithms and producing fewer false positives on confusing areas such as stems and surface blemishes [141]. Detection performance, however, varies by cultivar, with denser epidermal structures and higher activities of defense-related enzymes (PAL, POD) accounting for the stronger resistance observed in certain varieties.

Storage condition monitoring: Storage temperature and duration are critical environmental parameters affecting postharvest quality. Multi-task learning frameworks can build a single model that simultaneously outputs multiple related prediction targets. For yellow peaches stored under different conditions, a multi-target characteristic selection algorithm (DI-CARS) combined with a multi-task Wild Horse Optimizer–Support Vector Machine (WHO-SVM) model achieved 98.06% overall accuracy in simultaneously identifying storage condition (room temperature vs. low temperature) and storage time [142]. The DI-CARS method selected wavelengths that jointly characterized both storage condition and storage time, reducing computational cost by 66.67% compared to single-target approaches. The multi-task learning strategy exploits correlations among storage-related tasks—such as the intrinsic link between high-temperature storage and rapid quality deterioration—improving overall classification performance and generalization with small sample sizes while avoiding the overhead of training multiple independent models.

### 3.2. Seed Quality Testing

Hyperspectral imaging has emerged as a powerful non-destructive tool for seed quality evaluation, encompassing five major application categories: vigor assessment, variety identification, mechanical damage detection, mold contamination screening, and purity analysis. These applications leverage the sensitivity of HSI to biochemical and physical changes in seeds—including variations in moisture, protein, starch, lipids, and pigments—that correlate with germination performance, genetic identity, and storage quality.

Seed vigor assessment: Seed vigor, a key determinant of field emergence and crop establishment, is closely linked to physiological and biochemical changes during aging. The essence of vigor decline is ROS-triggered cascade damage, including lipid peroxidation, protein carbonylation, DNA damage, and mitochondrial disintegration [143]. As seeds age, lipid oxidation, protein denaturation, and membrane system damage lead to significant spectral shifts at specific bands (e.g., near 970, 1200, and 1450 nm). By employing spectral indices or machine learning models tailored to seed vigor, HSI can non-destructively predict germination rates, with models typically achieving high accuracy using SWIR spectral features. For instance, visible–near infrared HSI combined with SVM and Artificial Bee Colony optimization (ABC-SVM) achieved 100% accuracy in discriminating viable from non-viable watermelon seeds [144]. A recent study demonstrated that hyperspectral imaging combined with a residual Mamba convolutional neural network (RM1DNet) achieved 94.85% accuracy in classifying 20 maize seed varieties, outperforming traditional classifiers [145]. Similarly, NIR-HSI paired with deep convolutional neural networks (DCNN) has been applied to predict the vigor of corn seeds subjected to accelerated aging [59].

Variety identification and purity analysis: Differences in starch, protein content, and seed coat pigments among different seed varieties manifest as characteristic spectral features in the near-infrared region (1000–1300 nm). For example, NIR-HSI combined with stacked sparse autoencoder (SSAE) and CS-SVM achieved a classification accuracy of 95.81% for four maize seed varieties [60]. Similarly, VIS-NIR HSI combined with CARS-IRIV feature selection and SSA-SVM achieved 96.68% accuracy in identifying four red jujube varieties [146].

Mechanical damage detection: Mechanical damage during harvesting, threshing, and processing—such as cracking, skinning, and internal fissures—as well as environmental stresses like freezing and heat damage, adversely affect seed germination and storage longevity. HSI detects damage through changes in reflectance caused by altered surface structure and internal tissue exposure. Internal cracks invisible to the naked eye can be classified alongside intact, broken, and surface-cracked seeds with 99% accuracy [147]. These techniques enable automated, high-throughput sorting of damaged seeds in commercial seed processing lines.

Mold and fungal contamination screening: Fungal infection during storage induces chemical changes in seed components (e.g., lipid oxidation, protein degradation, starch hydrolysis) and alters surface reflectance in both visible and NIR/SWIR regions [148]. By identifying spectral absorption peaks or reflectance anomalies specific to infected areas, HSI enables early, non-destructive removal of contaminated seeds, avoiding the lengthy waiting periods of traditional culture methods, which is critical for preventing mycotoxin accumulation and ensuring food/feed safety.

Purity and authenticity analysis: Purity analysis encompasses not only the detection of off-type seeds within a variety but also the identification of admixture among different crop species (e.g., wild oats in wheat fields). By capturing subtle differences in seed coat pigments, starch granule morphology, and protein distribution, combined with multivariate statistical methods such as PCA, linear discriminant analysis (LDA), or deep learning, HSI can construct classification models [148]. This capability provides reliable technical support for seed quality regulation, variety authenticity verification, and market order maintenance. Physical dimensions—length and width—can be derived from the same hyperspectral data using a convex polygon algorithm, with relative error below 2% [149]. Sun et al. further demonstrated that combining spectral, morphological, and texture features from Vis-NIR HSI achieved 91.67% accuracy for rice origin identification [149]. Table 6 provides a comparative overview of these five application categories, summarizing their detection targets, primary spectral regions, modeling approaches, key advantages, and representative references. Figure 5 illustrates the steps of hyperspectral image acquisition, feature extraction, and classification of soybean seed quality using related techniques [150].

Staple grains and oilseeds require rapid, non-destructive quality screening for storage, processing, and trade. While compositional analysis (moisture, protein, starch) can be achieved through NIR-SWIR absorptions, the provided studies highlight HSI’s strength in detecting fungal contamination and storage pests. Raman hyperspectral imaging has emerged as a powerful tool for identifying *Aspergillus flavus* infection in peanuts, with SVM models achieving over 99% accuracy in binary classification and 88.9–92.4% accuracy for distinguishing three different fungal strains on single varieties [154]. Characteristic Raman peaks at 486 cm^−1^ (starch), 640 cm^−1^ (carbohydrates), and 1654 cm^−1^ (saturated fatty acids) enabled differentiation, as fungal metabolism alters kernel biochemistry. Additionally, HSI with chemometrics successfully detected storage mites (*Tyrophagus putrescentiae* and *Cheyletus eruditus*) in flour, achieving over 98% accuracy using ant colony optimization (ACO) for wavelength selection and artificial neural networks (ANN) for classification [155]. Reflectance differences among flour and the two mite species, particularly in the 640–730 nm range, allowed effective identification of these common pests, demonstrating HSI’s value for bulk grain storage management. X-ray CT combined with confocal microscopy has been used to monitor rice root tip cellular responses under compaction, providing a reference for internal seed defect detection [156].

Hyperspectral imaging technology has deeply penetrated into the whole chain of agricultural production through multi-dimensional and high-precision spectral analysis capabilities. Its successful application in physiological parameter inversion, adversity response diagnosis, environmental monitoring, and post-harvest management provides solid technical support for the precision and sustainable development of agriculture. In the future, it is necessary to further optimize the efficiency of data collection, reduce hardware costs, and promote technology standardization to accelerate its large-scale application in global agriculture.

### 3.3. Food Authenticity and Adulteration

Economically motivated adulteration and mislabeling demand rapid, non-destructive screening tools. HSI’s ability to capture subtle spectral differences makes it suitable for authenticating high-value foods and detecting chemical adulterants.

Authentication of high-value products: Seafood species substitution is a common fraud. Visible-NIR and SWIR HSI combined with LDA and RF have achieved 100% classification accuracy in distinguishing Atlantic cod from oilfish, *Dissostichus eleginoides*, and Greenland halibut; notably, frozen samples without thawing also attained 100% accuracy using the same LDA and RF models [157]. This discrimination relies on spectral fingerprints of myoglobin and fat composition, with characteristic absorption peaks at 417, 550, 575, and 650 nm related to heme pigments and myoglobin oxidation states.

For processed agricultural products, tea quality grading traditionally depends on subjective sensory evaluation. HSI offers an objective alternative. A ResNet-50 model incorporating a Two-Strategy Particle Swarm Optimization (TSPSO) algorithm and spatial information from the first three principal component images achieved 92.31% accuracy in classifying 13 quality grades of Huangshan Maofeng and Dianhong Gongfu teas [158]. Similarly, a PCA–TSPSO–ResNet-50 approach using the first three principal component images (cumulative contribution 99.88%) attained the same 92.31% test accuracy, confirming the potential of synergistic spatial–spectral fusion and hyperparameter optimization in tea quality grading [159]. Beyond grading, portable NIR spectroscopy coupled with chemometrics has been applied to quantify individual phenolic compounds (e.g., cianidanol, gallic acid, rutin) in congou black tea during fermentation, with correlation coefficients of prediction reaching 0.75–0.97 [160]. Complementarily, a solution-phase colorimetric sensor array combined with UV-Vis spectroscopy has achieved 100% accuracy in discriminating black tea fermentation degrees, with CARS-LSSVM models yielding R^2^ of 0.72–0.91 for catechins and theaflavins quantification [161].

Chemical adulteration detection: Illegal processing treatments introduce specific spectral signatures that can be captured by HSI. Saccharin-treated jujube, produced by soaking in saccharin solution, was successfully identified using NIR-HSI combined with VISSA-GWO-SVM, achieving 91.67% accuracy across four saccharin stress gradients [162]. The feature wavelengths selected by VISSA (161 wavelengths) were mainly distributed in the 500–680 nm, 680–760 nm, and 800–960 nm regions, corresponding to changes in chlorophyll, carotenoids, anthocyanins, and water. Similarly, for dried wolfberry, LS-SVM achieved 96.66% accuracy in quality grade classification after SG smoothing and SNV preprocessing [163], while sulfur-fumigated and Sudan Red-dyed wolfberries were identified with 96.7% accuracy using a VMD-CARS-SMA-SVM model [164]; GA-SVM optimization further improved the classification of adulterated wolfberry to 100% accuracy [165]. Beyond adulterant detection, NIR spectroscopy combined with LS-SVM and Si-PLS has been successfully applied to trace the geographical origin of both red and black goji berries (accuracies > 96.67% and 98.18%, respectively) and quantify bioactive compounds (flavonoids: R^2^ = 0.91; anthocyanins: R^2^ = 0.90) [166,167]. These examples highlight the versatility of HSI for detecting chemical adulteration in high-value medicinal and food products. Beyond intentional adulteration, pesticide residues pose significant safety risks. For chlorpyrifos residues on mulberry leaves, SPA-selected wavelengths (685 and 731 nm) combined with MLR yielded an R_p_ of 0.859 and an RMSEP of 38.789, and the resulting distribution maps successfully visualized residue heterogeneity, providing a novel approach for monitoring application safety [30]. Beyond HSI, SERS substrates (Au-Ag hollow cages) combined with CNN algorithms have achieved highly sensitive quantification of thiram and pymetrozine in tea, with detection limits of 0.286 and 29 ppb, respectively [168].

Mycotoxin detection: As a crossover target between natural toxins and adulterants, mycotoxins demand sensitive detection. Raman hyperspectral imaging provides molecular-specific vibrational information complementary to NIR absorption. For peanut kernels infected with different *Aspergillus flavus* strains, Raman hyperspectral imaging combined with SVM achieved high classification accuracy by leveraging characteristic peaks (e.g., 486, 640, 1654 cm^−1^) [154], offering a molecular fingerprint that complements conventional NIR approaches. Similarly, Raman spectroscopy combined with CARS-PLS has achieved high-precision prediction (R^2^ = 0.99) of aflatoxin B1 in pressed peanut oil [169]. Although Raman signals are weak and fluorescence-prone, its specificity for molecular backbone vibrations makes it valuable for complex food matrices. Alternatively, a colorimetric sensor array combined with swarm intelligence optimization (WOA-SVM) has achieved 95.83% accuracy for aflatoxin B1 detection in peanut oil [170].

Although tea tree oil fumigation effectively controls strawberry gray mold (95% AACPDi reduction at 600 μL/L), it causes softening, fading, and decreased phenolics, and it suppresses characteristic esters while increasing terpenes, significantly altering the flavor profile [171]. Therefore, HSI authenticity assessment should account for postharvest treatment history or integrate with multimodal fusion such as GC-IMS. Rapid identification of foodborne bacterial pathogens is a core challenge in food safety inspection. HSI provides a reagent-free, no-pre-enrichment method for detecting pathogens directly on agar plates or food surfaces; combined with PCA and PLS-DA, it enables accurate discrimination among multiple foodborne pathogens [14]. E-nose coupled with PSO-SVM has also achieved 98.95% accuracy in discriminating five foodborne bacterial pathogens at species and strain levels [172]. Physical foreign matters—plastics, metals, glass—pose safety risks comparable to biological and chemical contaminants, and their detection across meat, plant-based, and nut products has been surveyed [173]. A comprehensive review further consolidates the methodological framework for HSI-based foreign matter detection, covering hardware configurations, spectral preprocessing, feature engineering, and both machine learning and deep learning modeling strategies [174]. This approach can support high-throughput screening of multiple pathogens.

### 3.4. Meat, Egg and Processed-Food Quality

Animal-derived and processed foods feature complex matrices of protein, fat, and moisture, with quality changes often involving simultaneous structural and chemical evolution, demanding advanced HSI analysis.

Meat quality deterioration: Salt crystal size directly affects NaCl uptake and water activity in dry-cured pork, and HSI combined with CARS-PLS can effectively monitor the impact of crystal morphology on salt diffusion [175]. Freeze-thaw cycles disrupt muscle integrity, accelerating lipid and protein oxidation. Comparative studies on frozen pork showed that fluorescence HSI (F-HSI) with Gaussian Process Regression (GPR) achieved slightly superior TBARS prediction (R_p_^2^ = 0.9726, RMSEP = 0.0182 mg/kg) compared to vis-NIR HSI (R_p_^2^ = 0.9697, RMSEP = 0.0184 mg/kg) [176], a difference attributed to the sensitivity of fluorescence to oxidation-related fluorophores. F-HSI also enabled visualization of protein oxidation (carbonyls and total sulfhydryls), with VMD-MI-VIF-selected PLSR models achieving R_p_^2^ of 0.9275 and 0.9512, respectively [177]. Advanced data analysis further deepens mechanistic understanding: two-dimensional correlation spectroscopy (2D-COS) combined with 3D-CNN revealed the sequential order of spectral changes during lipid oxidation—decomposition of sulfmyoglobin and metmyoglobin preceded oxymyoglobin formation, and discoloration occurred before water loss—and achieved TBARS prediction without manual feature extraction (R_p_^2^ = 0.9214), a finding further validated by a lightweight 3D-CNN model using 2D-COS-selected bands [178]. Similarly, 2D-COS combined with a dual-branch CNN using bilinear pooling has achieved accurate TVB-N prediction (R^2^ = 0.9579) in pork, further validating the utility of 2D-COS for meat freshness assessment [179]. Beyond oxidation monitoring, soybean protein adulteration in minced chicken was identified with 98.1% accuracy using VGG16-SVM [180]. Multi-sensor fusion can break the information bottleneck of a single modality; the fusion of fluorescence HSI and E-nose (HFA-Net) improved pork freshness (TVB-N) prediction accuracy [38], and data fusion of spectral and GLCM texture features similarly enhanced prediction of lipid oxidation attributes (TBARS and peroxide value), yielding R_p_^2^ of 0.865 and 0.899, respectively [181].

Egg quality assessment: Haugh unit (HU), an international freshness index, was predicted non-destructively using XGBoost with IRIV-selected wavelengths in the 620–800 nm range (R_p_^2^ = 0.91, RMSEP = 4.64) [182]. CARS-LSSVM models predicted S-ovalbumin content in eggs during storage with R_p_^2^ = 0.918 and enabled visualization of its heterogeneous distribution [183]. Among superficially intact eggs, hyperspectral imaging reveals a distinct S-ovalbumin gradient, with elevated concentrations near the air cell, indicative of prior transient heat exposure. This biochemical alteration persists despite subsequent refrigeration, constituting a “thermal memory” effect that positions S-ovalbumin as a robust objective biomarker for cold-chain verification. Accordingly, this biomarker offers significant regulatory value in safeguarding food safety and combating the fraudulent misrepresentation of ambient-stored eggs as cold-chain products [184].

Complex processed foods: In heterogeneous multi-component foods such as spicy hotpot seasoning, HSI combined with a PSO-BPNN model achieved excellent prediction of capsaicin (R^2^ = 0.9942) and hydroxy-α-sanshool (R^2^ = 0.9939), with characteristic wavelengths identified in spectral regions associated with O–H, N–H, and C–H overtones [185]. The subsequent visualization of these pungent and numbing compounds within the matrix demonstrated the feasibility of HSI for quantifying trace active ingredients, opening possibilities for online quality control of condiments and prepared dishes. Recent advances in HSI-based foreign matter detection in meats, plant foods, and nut products have been reviewed [186]. HSI has also been employed to visualize the distribution of moisture, reducing sugars, and chewiness in bread bolus during oral processing [187]. VIS-NIR HSI successfully identified homochromatic foreign materials in cut tobacco with 100% classification accuracy [186].

## 4. Challenges and Development Directions

### 4.1. Core Bottlenecks in Field Deployment

Across the studies reviewed above, several recurring limitations become apparent when HSI systems are transferred from controlled experiments to field or production environments. These include incomplete metadata and calibration reporting, reduced model performance across sites and instruments, hardware and environmental constraints, and difficulties in translating model outputs into operational decisions. This chapter discusses four recurring bottlenecks and outlines possible responses.

Lack of data standardization: The global agricultural HSI research community generates highly fragmented data, and this fragmentation pervades every stage of the data lifecycle. Underlying storage formats are mutually incompatible, with ENVI header/binary format, HDF5, and various proprietary binary formats coexisting. Metadata specifications remain severely inadequate, as critical parameters—including acquisition time, sun–target–sensor geometry, sensor model, and integration time—are frequently recorded selectively or omitted entirely. Preprocessing heterogeneity is not, in itself, evidence of poor practice. Different optical architectures, detector materials, acquisition modes, spectral ranges, illumination geometries, and deployment platforms inevitably require different calibration and correction procedures. The more serious problem is that these instrument characteristics are often not reported in sufficient detail, and there is no widely accepted set of minimum calibration requirements for each acquisition architecture. As a result, cross-study comparison and data reuse remain difficult even when the underlying data are otherwise comparable. Annotation standards also lack uniformity, with reference values derived from either laboratory chemical assays or field-based phenotyping platforms, while sample sizes and accuracy metrics are defined and reported at each team’s discretion. This fragmentation originates from a persistent absence of international standards: although ISO and IEC have issued general remote sensing guidelines, a standard specifically tailored to agricultural HSI—covering data formats, metadata, quality control, and interoperability—remains unavailable, hindering the formation of a collaborative ecosystem among instrument manufacturers, service providers, and researchers.

Several consequences follow. Spectral data acquired from different projects, instruments, and sites cannot be robustly fused, limiting the scale and diversity of training datasets and depriving deep learning models of the large, heterogeneous corpora they require. Cross-comparison of model performance across studies becomes unfounded, as a reported accuracy of 95% in one investigation and 90% in another may stem from fundamentally different benchmarks. More critically, the ambiguity surrounding raw data acquisition, processing, and annotation renders independent third-party verification nearly impossible, eroding the foundational principle of scientific reproducibility. Consequently, the agricultural HSI field still lacks a widely recognized benchmark database comparable to ImageNet in computer vision. Although several pioneering datasets have been introduced—such as Agro-HSR [188] for image reconstruction, the Heilongjiang Benchmark Dataset [189] for large-scale crop classification, the Rice Seed dataset [190] for seed identification, HSI Agri-Food-Anomaly [191] for foreign object detection, and the Handheld Hyperspectral Imaging Dataset [192] for simulated production-line stress scenarios—these isolated efforts remain far from sufficient to establish a unified “data contract,” and the absence of systematic standards remains an obstacle to large-scale deployment.

Insufficient Model Generalization: High-performance models frequently reported in the literature are often built under highly idealized conditions—data acquisition is restricted to a single geographic location, a single growing season, a single crop cultivar, and a single finely calibrated sensor. When such models are deployed to scenarios outside the training distribution, however, their performance frequently degrades sharply. A representative case is the prediction of apple SSC, where a near-infrared calibration model applied to samples harvested in different years exhibited a dramatic increase in root mean square error of prediction (RMSEP) from 0.547% to 1.716%; this discrepancy could only be partially mitigated by calibration transfer methods such as Slope/Bias correction [135]. These effects arise from several coupled sources, including sensor-to-sensor differences in spectral response functions, inconsistencies in radiometric calibration, BRDF effects that vary with illumination and viewing geometry, and atmospheric heterogeneity in airborne or spaceborne data.

From the perspective of machine learning theory, this constitutes a severe out-of-distribution (OOD) generalization problem: the covariate distributions (spectral features, environmental parameters, biological states) represented in the training data fail to capture the nearly infinite combination of possibilities encountered in real-world settings. Zhong et al. [193] demonstrated that without rigorous spectral feature adaptation, classification accuracy drops significantly when transferring HSI models across different sensors and scenes, highlighting the domain shift problem in cross-scenario deployment. For instance, UV-Vis spectroscopy combined with one-class support vector machines (OC-SVM) has been successfully applied to detect syrup-adulterated honey [194]. Although these strategies have shown promise in laboratory validation, the gap between controlled settings and complex field environments remains substantial. While Fourier transform infrared spectroscopy (FTIR) has proven effective in distinguishing pure honey from samples adulterated with various syrups (e.g., agave, corn, invert, maple, and rice), the model performance is heavily matrix-dependent and may deteriorate considerably when encountering floral origins not represented in the training set [195]. Beyond syrup adulteration, NIR spectroscopy has also been applied to quantify antioxidant properties (phenolics, flavonoids, FRAP) and discriminate honey floral origins, with LDA achieving 100% classification accuracy in Sudanese honeys [196].

Hardware costs and environmental adaptability: The high cost of sensors remains a major constraint. Currently, mainstream high-performance focal plane arrays (FPAs) are predominantly based on cooled mercury cadmium telluride (MCT) or indium gallium arsenide (InGaAs) technologies. MCT detectors, though capable of covering a broader spectral range (up to 14 μm), suffer from difficult material growth, poor uniformity, and the necessity of deep cooling to suppress dark current, which directly drives up system complexity and cost [197]. InGaAs detectors perform well in the 0.9–1.7 μm band, but their InP substrates are expensive, and extending the response beyond 1.7 μm typically requires more complex detector structures, which increases fabrication difficulty and cost [198,199]. Although novel low-cost sensors based on colloidal quantum dots (CQDs) promise to reduce costs by an order of magnitude, their quantum efficiency, response speed, and long-term stability still lag significantly behind mature InGaAs technology [200,201]. For UAV-borne HSI systems, additional challenges arise from the “impossible triangle” among payload, endurance, and spectral quality. A typical high-performance pushbroom imager (e.g., the Hy-Spex series) with its stabilization gimbal often weighs over 3 kg, severely curtailing the effective flight time of consumer-grade or light agricultural UAVs [202]. Snapshot or filter-wheel systems developed for weight reduction usually sacrifice spectral resolution, spatial resolution, or signal-to-noise ratio, making it difficult to acquire high-quality data that meet the requirements of precision agricultural diagnostics [203].

Field environmental factors further degrade data quality. Under laboratory conditions, constant temperature and humidity can maintain high wavelength stability and radiometric calibration accuracy, but temperature fluctuations in the field can cause minute deformations of dispersive elements such as gratings or prisms, leading to temperature-induced wavelength drift [204]. Humidity changes affect the transmittance of optical components, dust and water vapor contaminate lens surfaces, and onboard light-source intensity decays with battery status and temperature—problems largely absent in the laboratory that collectively induce unpredictable variations in detector responsivity. Temperature also affects the biological state of the sample itself: elevated temperature shifts glucose metabolism from extracellular oxidation to intracellular phosphorylation, fundamentally altering the organism’s spectral phenotype [205]. Even when sensors are successfully deployed, long-term radiometric calibration stability remains a persistent issue; performing cumbersome laboratory-level recalibration before each flight is impractical for routine agricultural operations, while scene-based alternative methods such as pseudo-invariant calibration sites or empirical line approaches have limited reliability in complex farmland scenarios. BRDF anisotropy adds another layer of fundamental complexity: crop canopies are not ideal Lambertian surfaces, and their reflectance characteristics depend strongly on solar incidence angle and sensor viewing geometry—spectral reflectances acquired from the same field at different times or geometries cannot be directly compared, which can seriously interfere with physiological parameter retrieval based on spectral indices and may even mask true biotic stress signals [204]. The interplay of these hardware and environmental constraints means that SWIR-HSI deployment is currently dominated by well-funded research institutions and large agribusinesses, effectively excluding ordinary smallholders and small-to-medium food processors.

Barriers to user adoption: The operation, data interpretation, and decision translation of HSI systems require interdisciplinary expertise spanning spectroscopy, machine learning, crop science, and food engineering—a threshold that directly excludes small-scale producers, who constitute the majority of the global agricultural workforce. These producers typically have limited awareness of HSI capabilities, often perceiving the equipment as overly complex, expensive, and difficult to integrate into routine management workflows [206,207]. The conventional capital expenditure model, requiring one-time acquisition of high-value instruments, is severely mismatched with the limited financial capacity of smallholders; although service-based alternatives such as area-based UAV HSI services or cooperative equipment sharing are theoretically more feasible, they remain at an early developmental stage in practice, lacking mature business models and extensive coverage networks [208]. Meanwhile, in the absence of clear data ownership frameworks and transparent consent protocols, the collection of high-resolution field data readily raises privacy concerns, further eroding farmers’ trust.

However, the most fundamental obstacle lies in the “last mile” translational gap between model outputs and actionable decisions. Ordinary farmers or food processing line operators have no need to know the number of latent variables in a PLSR model or the kernel size of a CNN; what they truly require are concise, unambiguous, and context-specific action directives, such as “nitrogen deficiency in the northeast corner of the field, recommend top-dressing 15 kg/mu of urea” or “the SSC of this batch of apples is below 12%, recommend redirecting to processing use” [209]. The key intermediary in bridging this gap—the agronomist—though deeply knowledgeable about crop growth patterns, often lacks professional training in spectral data interpretation; conversely, data scientists proficient in algorithms have limited understanding of specific agronomic practices and crop physiological responses. This bidirectional knowledge divide can only be overcome through institutionalized interdisciplinary joint training.

### 4.2. Solution Pathways and Technology Outlook

Data standardization remains a foundational requirement. Data standardization involves three types of requirements: common metadata, architecture-specific minimum calibration protocols, and environment-specific preprocessing guidelines. Not all corrections are universally required; each scenario imposes its own minimum set. The GRSS has initiated IEEE PROJECT 4001 to standardize the definition and reporting of instrument specifications [210], and this effort can be extended to agricultural applications.

With this three-layer distinction in mind, the specific actions remain threefold:

(1) International academic organizations such as the IEEE Geoscience and Remote Sensing Society (GRSS), together with major HSI instrument manufacturers (e.g., Headwall, Specim) and leading agricultural research institutions (e.g., CIMMYT, IRRI), should formulate community-consensus format specifications for agricultural hyperspectral data. This includes the common metadata requirements described above.

(2) The community should also build and maintain open benchmark databases for agricultural HSI, covering major crops (rice, wheat, maize, soybean), contrasting soil types, and key food categories, with multi-year, multi-site, and multi-sensor observations collected under FAIR principles. Agro-HSR is an early example of such a resource [188], and the analysis of 18,730 soil samples from Parana, Brazil [211], illustrates how large standardized spectral datasets can support predictive models. Similar efforts are needed for crop phenotyping and pest/disease monitoring.

(3) Journals should require independent test-set validation as a condition for publication. A model developed on one farm in one year may perform poorly in another region or season [212]. Requiring cross-site and cross-year validation would push the community toward generalizable methods rather than leaderboard performance, and would give commercial users a more reliable basis for selection.

At the instrument level, minimum calibration requirements should be architecture-specific. Pushbroom systems should report wavelength calibration, smile/keystone characterization, and line-registration procedures; wavelength-scanning or staring systems should document spectral tuning accuracy, inter-band registration, and illumination stability; snapshot systems should report spatial–spectral calibration, reconstruction procedures, and crosstalk characterization. For laboratory HSI, dark/white-reference and flat-field correction are generally sufficient for radiometric normalization, whereas field and remote-sensing systems may additionally require geometric, illumination, BRDF, or atmospheric corrections depending on the acquisition conditions.

These actions are logically ordered rather than independent. Without a standardized data foundation, generalization algorithms cannot be fairly compared, hardware cost reduction lacks demand-side drivers, and business-model innovation lacks data exchange and interoperability. The development of cross-platform interface standards can facilitate seamless device connectivity, enhance data-sharing efficiency and interoperability, and lay the groundwork for large-scale technology deployment. The construction of shared spectral libraries—for example, a spectral library for leaf disease hyperspectral imaging that contains pure spectral “fingerprints” of multiple pathogens across different crops and infection stages [213], which, after rigorous atmospheric correction and radiometric calibration, can serve as endmembers for quantitative disease severity inversion or as a pretraining knowledge base for machine learning to reduce annotation dependence—represents a concrete step in this direction. This approach is corroborated by the LUCAS project’s mid-infrared soil spectral library, which demonstrates that a high-quality reference library can effectively support multi-scale applications ranging from regional surveys to field-level precision fertilization [214].

Breakthroughs in Model Generalization: The key to bridging the performance gap between laboratory and field conditions lies in enabling models to handle variations not encountered in the training data, rather than overfitting to observed variations. At its core, this gap stems from domain shift—a compound effect of sensor differences, illumination changes, atmospheric interference, and vegetation structural diversity. Several technical directions are relevant:

(1) Adversarial domain adaptation with disentangled representation, which aligns source- and target-domain distributions in the feature space to reduce cross-scene discrepancies without requiring labeled target-domain data. This is exemplified by combining the PROSPECT-D physical model with an adversarial ResNet for reliable estimation of key traits such as chlorophyll across different ecosystems [215], as well as by the S4DL framework, which refines decoupling by separating domain-specific and domain-invariant features [216].

(2) Physics-informed neural networks (PINNs), which embed radiative transfer models (e.g., PROSPECT, SAIL) as constraints or priors into the network, thereby ensuring that predictions under extreme conditions (e.g., severe drought or exceptionally high chlorophyll) remain physically plausible. This marks a pivotal evolution from purely data-driven approaches to mechanism–data dual-driven paradigms. Recent studies have applied PINNs to solve the radiative transfer equation [217], coupled RTM features for cross-scale nitrogen concentration estimation [218], and directly inverted PROSAIL parameters from Sentinel-2 imagery [219]. Beyond radiative transfer models, process-based crop growth models (e.g., SWAP and WOFOST) have also been successfully parameterized for root water uptake and biomass partitioning in winter wheat, rapeseed, and summer maize, offering a complementary physical backbone for data-driven agricultural sensing [220,221,222].

(3) Self-supervised pre-training with masked autoencoders (MAE), which leverage massive unlabeled spectral data to train a general-purpose feature backbone, followed by fine-tuning with limited labeled samples, enhancing few-shot generalization. For instance, SST-MAE, designed for plant hyperspectral time series, learns genotype–phenotype relationships without trait annotations [223], offering a scalable pathway toward foundational model training in this domain.

These three pathways are synergistic and mutually reinforcing: self-supervised pre-training provides generic representations, physical constraints ensure extrapolation reliability, and domain adaptation bridges discrepancies across heterogeneous data sources. These directions are complementary and may facilitate laboratory-to-field transfer.

Hardware and computing cost reduction: CQD-based SWIR detectors are being explored as potentially lower-cost alternatives to conventional InGaAs devices because they can be processed from solution and integrated with CMOS readout circuits. However, their long-term stability, uniformity, dark current, and device maturity remain important limitations. Moreover, the photosensitive layer can be extended to cover the SWIR and even mid-wave infrared (MWIR) ranges [224], with performance increasingly approaching that of commercial InGaAs devices [225,226]. Concurrently, the adoption of multi-band LED active illumination as a substitute for sunlight eliminates the interference of ambient light variations on spectral measurements, while the fixed source-detector geometry simplifies radiometric calibration procedures, rendering one-time factory calibration a practically feasible solution.

At the computing level, the combined application of knowledge distillation (transferring knowledge from large models to smaller ones), model pruning, and quantization can reduce model size and computational demand, facilitating real-time inference on edge devices [227]. Selected informative wavebands can also guide the design of task-specific multispectral sensors, potentially reducing data volume and hardware cost for narrowly defined applications [228]. This approach can reduce deployment costs in specific applications.

Business model innovation: Shifting the economic burden of technology deployment upstream from the user side represents a critical leverage point for bridging the adoption gap. Two practical service models merit consideration:

(1) The Software-as-a-Service (SaaS) model—users upload spectral data via mobile devices or connect to lightweight acquisition terminals, while cloud-based AI models automatically perform analysis and return visualized agronomic recommendations, with fees charged per use or per unit area. Studies have shown that cloud-based agricultural pest and disease prediction platforms are already capable of real-time monitoring of crop growth status and meteorological conditions [229]; similarly, SaaS services for satellite remote sensing data have demonstrated that cloud platforms can efficiently process high-resolution imagery and generate standardized products [230], providing a transferable pathway for cloud-based HSI data processing.

(2) The shared-service model—cooperatives or specialized service organizations procure UAV-borne or handheld HSI equipment collectively and provide periodic field scouting and diagnostic services to multiple smallholders within a region, thereby spreading per-unit costs through scaled operations. Empirical studies indicate that agricultural socialization services facilitate the adoption of sustainable technologies among smallholders [231] and effectively promote sustainable growth in food production; optimizing service supply chain scheduling can further align farmer demand with service resource allocation [232].

## 5. Conclusions

This review summarizes HSI applications from crop and soil sensing to postharvest quality and food safety. Under controlled or semi-controlled conditions, HSI has produced useful results for chlorophyll and nitrogen estimation, stress detection, soluble-solids prediction, authenticity assessment, and contaminant screening. The main limitation is not whether these attributes can be sensed, but whether calibration procedures and predictive models remain reliable when instruments, seasons, locations, or operating conditions change. Wider deployment will therefore depend on more transparent reporting of instrument and calibration parameters, stronger external validation, sensing systems matched to specific tasks, and simpler workflows for end users. These requirements are likely to determine which HSI applications move from experimental demonstrations to routine agricultural and food-quality monitoring.

## Figures and Tables

**Figure 1:**
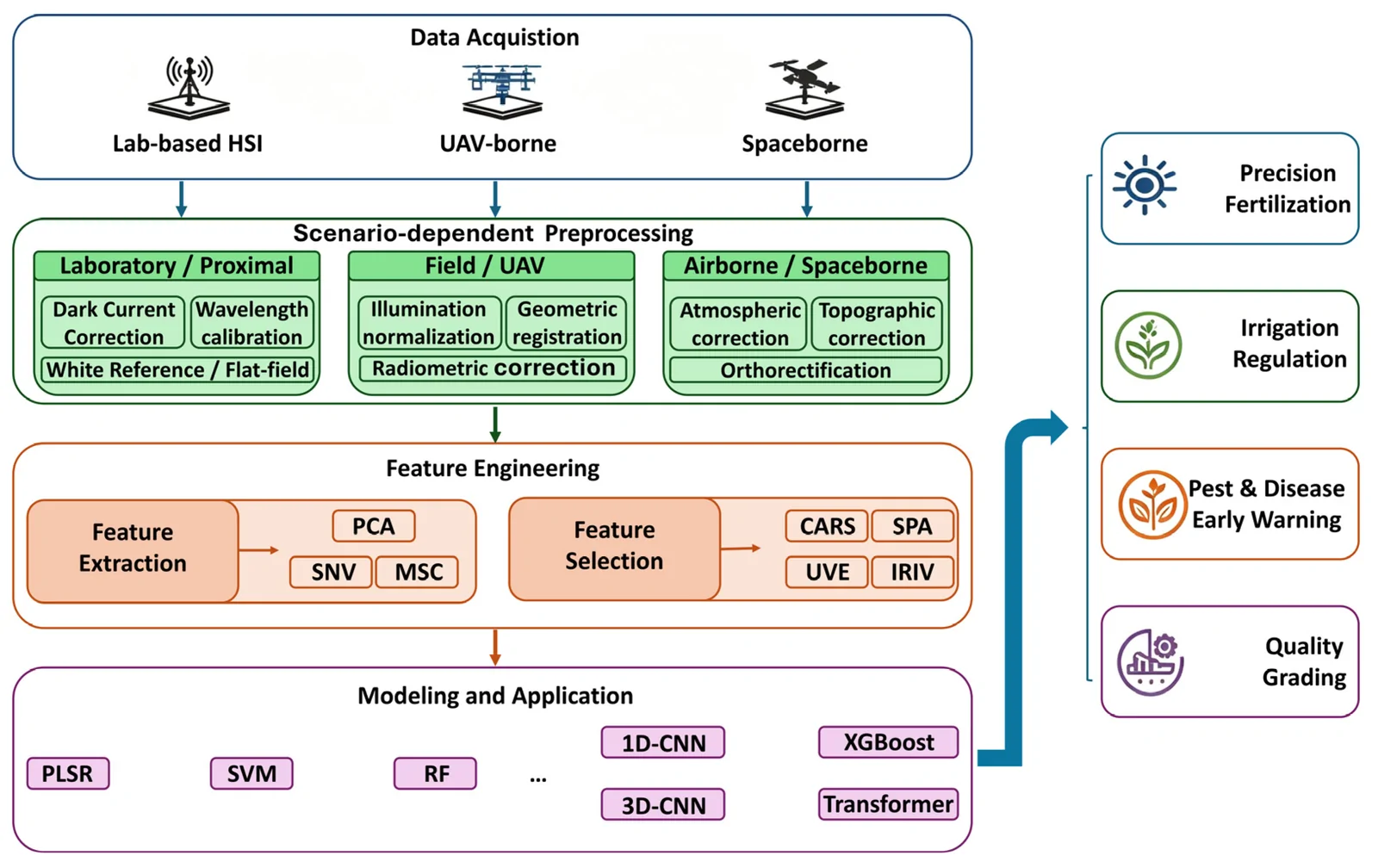
A generalized and scenario-dependent workflow for hyperspectral imaging. The framework distinguishes acquisition environments and instrument architectures (top layer), scenario-specific calibration and preprocessing requirements (middle layer), and a common data-analysis backbone (bottom layer). The preprocessing block is shown in a simplified form; atmospheric correction is only applicable to airborne and spaceborne data, or to field applications when atmospheric path effects are non-negligible, whereas laboratory and proximal systems require dark-current, white-reference, and flat-field corrections. The workflow is not a universal protocol but a common analytical backbone that adapts to specific instruments and deployment scenarios.

**Figure 2:**
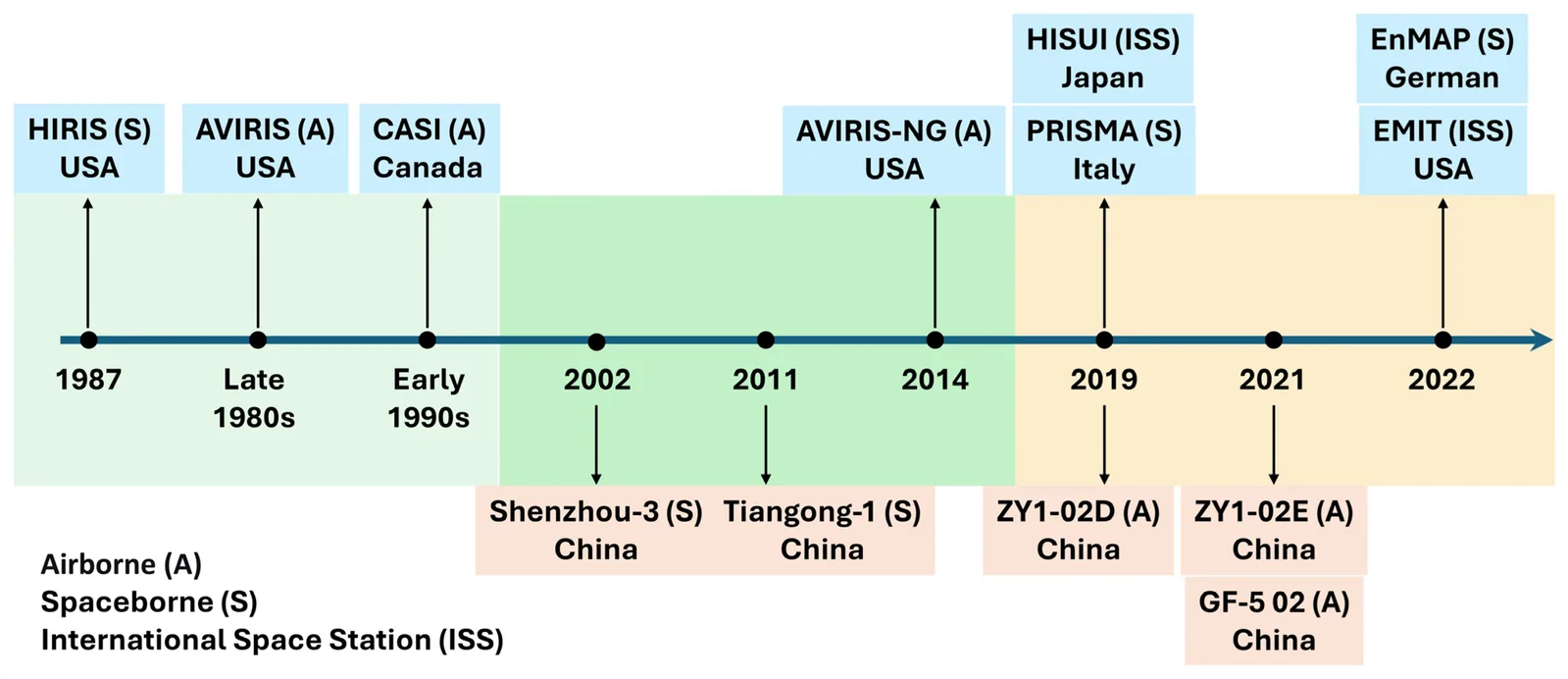
Timeline of representative international hyperspectral imaging missions (1980s–2020s).

**Figure 3:**
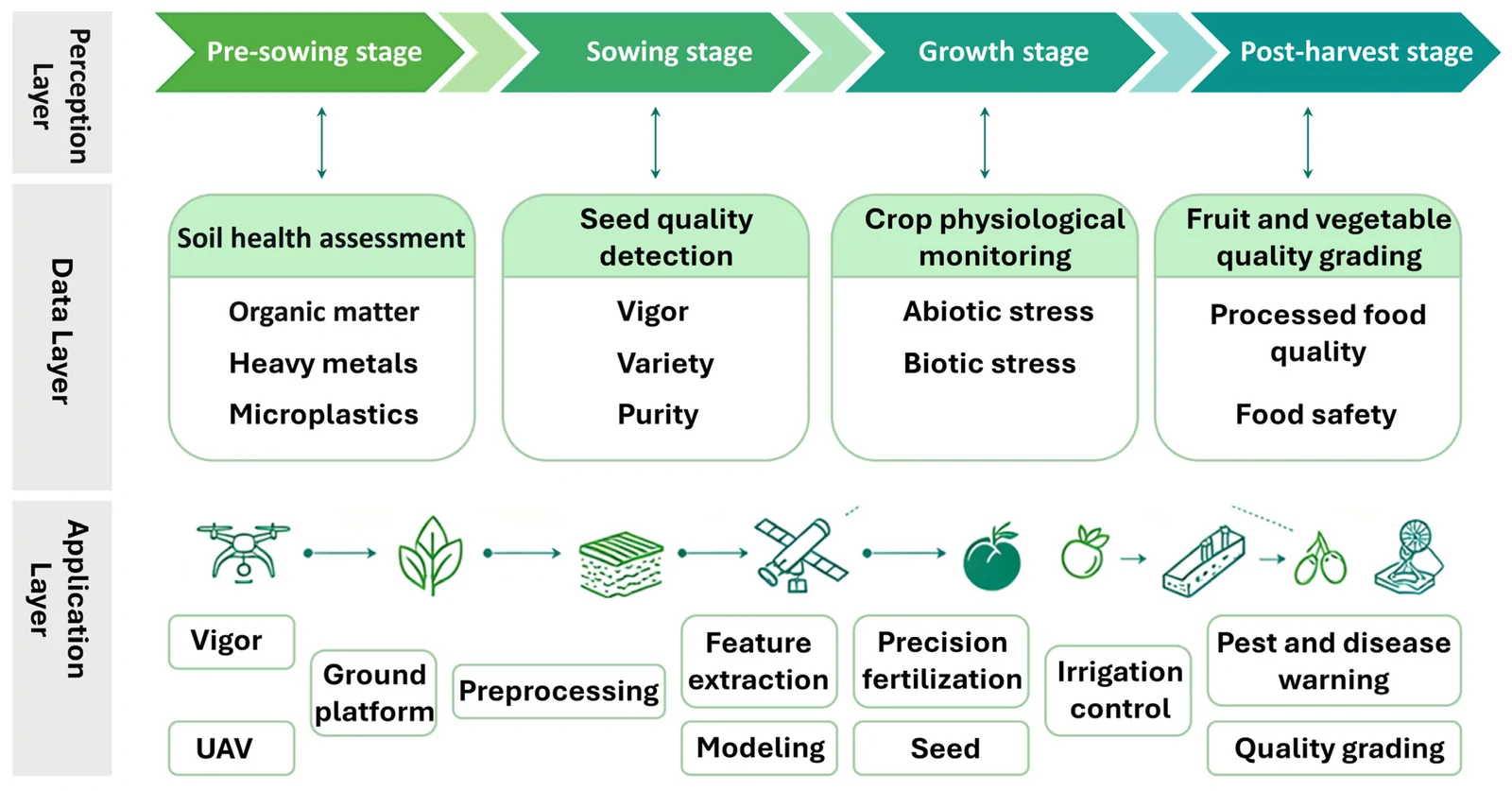
Hyperspectral imaging application scenarios in agriculture.

**Figure 4:**
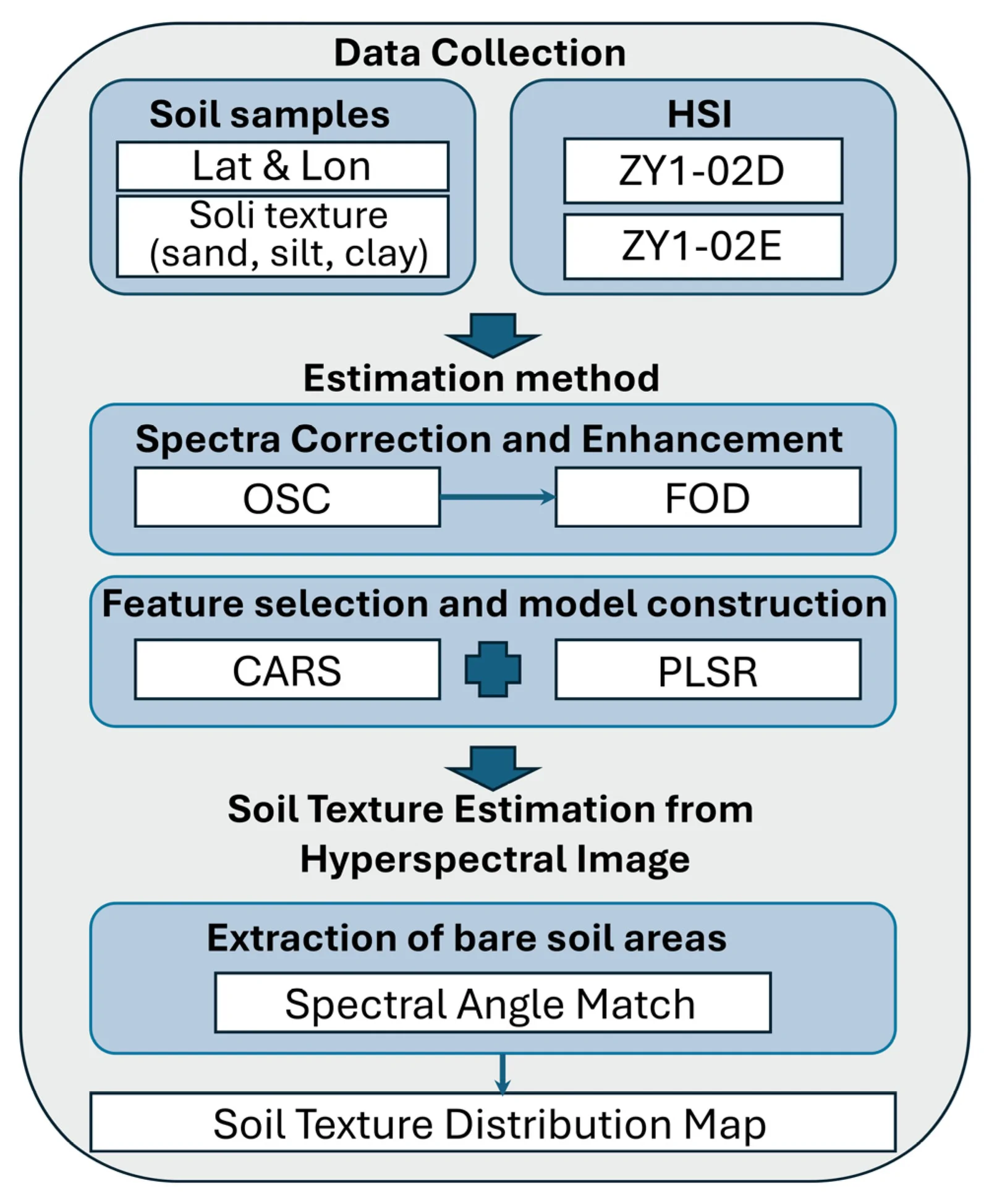
Soil texture detection process based on hyperspectral imaging. OSC, orthogonal signal correction; FOD, fractional-order derivative; CARS, competitive adaptive reweighted sampling; PLSR, partial least-squares regression. Arrows indicate the processing sequence; colors are used for visual distinction only. Adapted from Ref. [118] under the CC BY 4.0 license.

**Figure 5:**
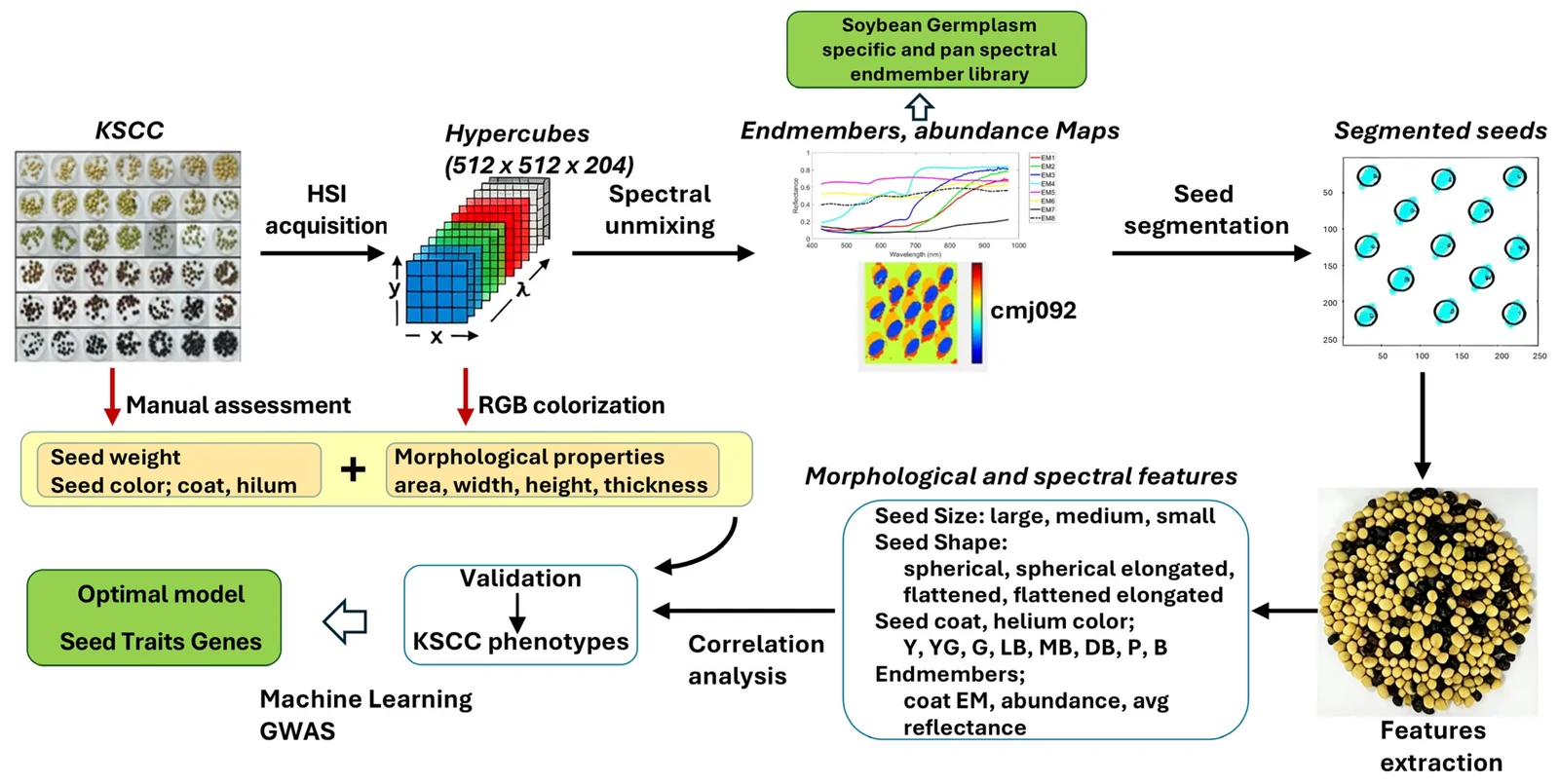
Key steps in hyperspectral image acquisition, feature extraction, and classification of soybean seed germplasm using the SUNSeT toolbox integrated with genome-wide association studies (GWAS). KSCC, Korean Soybean Core Collection; HSI, hyperspectral imaging; RGB, red–green–blue; GWAS, genome-wide association study. Arrows indicate the workflow; colors are used for visual grouping. Adapted from Ref. [150] under the CC BY 4.0 license.

**Table 1:** Representative acquisition and spectral-encoding architectures for HSI. These categories describe different aspects of spatial/spectral acquisition and are not necessarily mutually exclusive. Fourier-transform and computational approaches describe spectral-encoding strategies that may overlap with scanning or snapshot acquisition modes.

Imaging Modality	Instantaneous Sampling Manifold	Temporal Coherence	SNR Characteristics
Whiskbroom	0D point	Low (sequential point scanning)	High (long dwell time per pixel)
Pushbroom	1D line [22]	Low (temporal skew between lines) [22]	High (long dwell time equivalent to cross-track pixels)
Staring/wavelength-scanning	2D scene at one band	Medium (sequential band tuning)	Medium
Snapshot	2D (full scene encoding) [23]	High (single integration, no scanning) [23]	Moderate (inherent spatial–spectral–throughput trade-off)
FTIR	2D (spatial), spectral via temporal interferogram [24]	Medium (requires scanning optical path difference) [24]	High (multiplex and throughput advantages) [24]
Computational Spectral Imaging	Encoded 2D	Variable (snapshot for CASSI [25], others vary)	Algorithm-dependent, can be enhanced by deep learning

**Table 2:** Comparison of RGB, Multispectral, and Hyperspectral Imaging Technologies.

Imaging Type	Data Dimensionality	Typical Platforms	Wavelength Range	Number & Type of Bands	Typical Detector and Spectral-Selection Architecture
RGB Imaging	2D spatial (x, y), 3 values per pixel (R/G/B)	Consumer-grade devices (smartphones, drones, surveillance systems)	Approximately 400–700 nm, depending on the adopted convention and sensor specification	3 broad bands (red, green, blue)	Si CMOS/CCD detectors with color-filter arrays
Multispectral Imaging	3D data cube (x, y, λ), limited and discontinuous number of bands	Satellite remote sensing (Sentinel-2, Landsat), agricultural drones [40], portable multispectral imaging systems [41]	400–2500 nm (covering parts of VNIR to SWIR; sensor-dependent)	4–15+ selected discrete narrow bands [40]	Si CMOS/CCD or InGaAs detectors depending on spectral range, combined with discrete filters, filter arrays, filter wheels, or multichannel optics
Hyperspectral Imaging	Full 3D data cube (x, y, λ), each spatial pixel has a near-continuous spectral curve [42]	Satellites (AVIRIS), manned aircraft, light UAVs, ground-based laboratories	400–2500 nm (VNIR–SWIR), continuous coverage [43]	100–300+ continuous narrow bands (full-spectrum continuous coverage)	CMOS/CCD, InGaAs, or other detector arrays depending on spectral range, combined with grating/prism dispersion, tunable filters, interferometric, snapshot, or computational spectral encoding

**Table 3:** Representative data-analytics approaches for HSI.

Method Category	Representative Methods	Key Strengths	Suitable Conditions
Chemometrics	PCA, PLSR, LDA	Small-sample, interpretable	Moderate datasets, quantitative analysis
Conventional ML	SVM, RF, XGBoost, KNN	Nonlinear, less feature engineering	Small-to-moderate sample sizes
Deep learning	1D/2D/3D-CNN, CNN-RNN, Transformer	Spectral–spatial joint learning, end-to-end	Large labeled datasets, complex spatial–spectral patterns
Emerging learning	Self-supervised, MAE, domain adaptation, transfer learning	Few-shot, cross-scenario generalization	Limited labels, cross-sensor/year/region transfer

**Table 4:** Comparison of representative red-edge vegetation indices for chlorophyll estimation.

Index Name	Primary Use	Requires Red-Edge Band?	Key Characteristic (from the Review)
Red-edge Normalized Difference Vegetation Index (NDVI705)	Chlorophyll content estimation	Yes	Replaces the conventional red band with 705 nm to mitigate saturation at high chlorophyll levels; ρ705 maintains a wider dynamic range [70]
Modified Chlorophyll Absorption Ratio Index (MCARI)	Chlorophyll-a estimation; sensitive to soil background	Yes (700 nm)	Quantifies the relationship between chlorophyll absorption depth at 670 nm and the green peak; originally designed to be highly sensitive to LAI [71]
Red-edge Chlorophyll Index (CIred-edge)	Chlorophyll content (commonly used with Sentinel-2)	Yes	Uses a simple ratio of NIR and red-edge bands; exhibits a wider linear response range than NDVI-based indices [70]
TCARI/OSAVI	Minimizes soil background effects; suitable for sparse canopies	Yes	Normalizes the chlorophyll absorption signal (TCARI) by the soil-adjusted canopy structure signal (OSAVI), decoupling chlorophyll content from LAI and soil brightness [71]
Red-Edge Inflection Point (REP)	Chlorophyll content (more sensitive at high concentrations)	Yes (680–750 nm)	Robust to overall spectral scaling or shifts caused by illumination intensity and sensor gain; strongly correlated with leaf and canopy chlorophyll content [72]

**Table 5:** Summary of hyperspectral imaging and machine learning studies for LNC and soil nitrogen mapping in major crop systems.

Crop	Key Bands/Wavelengths	Model	Reference
Summer maize	Red-edge region (700–750 nm)	Red-edge parameters/regression model	[77]
Winter wheat	Red-edge parameters	Red-edge-based estimation model	[78]
Tomato	Around 1510 nm	Machine learning model	[67]
Rice	Red edge, 1510 nm, 1940 nm, etc.	Deep learning model	[80]
Soybean	Red-edge and SWIR bands	MLR model	[81]
Grassland	1510 nm	Machine learning	[79]

**Table 6:** Comparison of five major application categories of hyperspectral imaging in seed quality evaluation.

Application Category	Detection Targets/Indicators	Primary Spectral Regions	Common Modeling Approaches	Representative References
Seed vigor assessment	Germination rate, vigor index, aging degree; lipid peroxidation and protein carbonylation markers	NIR/SWIR (around 970, 1200, 1450 nm)	Grouped fusion strategies, SAM-GAN, SWIR-based PLSR	[151]
Variety identification	Variety authenticity, haploid/diploid discrimination	NIR (1000–1300 nm)	One-class learning, self-supervised learning, ensemble deep learning	[152]
Mechanical damage detection	Cracks, skinning, fissures, freezing/insect damage	Full spectrum (400–2500 nm)	DCNN, 1D-CNN-BiLSTM	[153]
Mold and fungal contamination screening	Fungal infection, mycotoxin risk	VIS/NIR/SWIR	SVM, spectral anomaly detection, Raman HSI	[148]
Purity and authenticity analysis	Off-type seed proportion, species admixture	Full spectrum (400–2500 nm)	PCA, LDA, deep learning (CNN)	[148]

## Data Availability

No new data were created or analyzed in this study. Data sharing is not applicable to this article.

## References

[1] Osman M.A., Onono J.O., Olaka L.A., Elhag M.M., Abdel-Rahman E.M. (2021). Climate variability and change affect crops yield under rainfed conditions: A case study in Gedaref State, Sudan. Agronomy.

[2] Chauhdary J.N., Li H., Pan X., Zaman M., Anjum S.A., Yang F., Akbar N., Azamat U. (2025). Modeling effects of climate change on crop phenology and yield of wheat–maize cropping system and exploring sustainable solutions. J. Sci. Food Agric..

[3] Chauhdary J.N., Li H., Ragab R., Rakibuzzaman M., Khan A.I., Zhao J., Akbar N. (2024). Climate Change Impacts on Future Wheat (*Triticum aestivum*) Yield, Growth Periods and Irrigation Requirements: A SALTMED Model Simulations Analysis. Agronomy.

[4] Hasegawa T., Sakurai G., Fujimori S., Takahashi K., Hijioka Y., Masui T. (2021). Extreme climate events increase risk of global food insecurity and adaptation needs. Nat. Food.

[5] Coulibaly J.Y., Gbetibouo G.A., Kundhlande G., Sileshi G.W., Beedy T.L. (2015). Responding to Crop Failure: Understanding Farmers’ Coping Strategies in Southern Malawi. Sustainability.

[6] He W., Liu Y., Sun H., Taghizadeh-Hesary F. (2020). How Does Climate Change Affect Rice Yield in China?. Agriculture.

[7] Islam D.I., Rahman A., Sarker M.S.R., Luo J., Liang H. (2021). Factors affecting farmers’ willingness to adopt crop insurance to manage disaster risk: Evidence from Bangladesh. Int. Food Agribus. Manag. Rev..

[8] Rosegrant M.W., Sulser T.B., Dunston S., Mishra A., Cenacchi N., Gebretsadik Y., Robertson R., Thomas T., Wiebe K. (2024). Food and nutrition security under changing climate and socioeconomic conditions. Glob. Food Secur..

[9] Liang Y., Li Z., Shi J., Zhang N., Qin Z., Du L., Zhai X., Shen T., Zhang R., Zou X. (2025). Advances in Hyperspectral Imaging Technology for Grain Quality and Safety Detection: A Review. Foods.

[10] Cheng J., Sun J., Yao K., Xu M., Tian Y., Dai C. (2022). A decision fusion method based on hyperspectral imaging and electronic nose techniques for moisture content prediction in frozen-thawed pork. LWT-Food Sci. Technol..

[11] Fakhlaei R., Babadi A.A., Sun C., Ariffin N.M., Khatib A., Selamat J., Zou X. (2024). Application, challenges and future prospects of recent nondestructive techniques based on the electromagnetic spectrum in food quality and safety. Food Chem..

[12] Shi Y., Wang Y., Hu X., Li Z., Huang X., Liang J., Zhang X., Zheng K., Zou X., Shi J. (2023). Nondestructive discrimination of analogous density foreign matter inside soy protein meat semi-finished products based on transmission hyperspectral imaging. Food Chem..

[13] Cheng J., Zhang G., Abdulla W., Sun J. (2024). Advancing fraud detection in New Zealand Mānuka honey: Integrating hyperspectral imaging and GANomaly-based one-class classification. Food Biosci..

[14] Bonah E., Huang X., Aheto J.H., Osae R. (2019). Application of Hyperspectral Imaging as a Nondestructive Technique for Foodborne Pathogen Detection and Characterization. Foodborne Pathog. Dis..

[15] Yao K., Sun J., Zhou X., Nirere A., Tian Y., Wu X. (2020). Nondestructive detection for egg freshness grade based on hyperspectral imaging technology. J. Food Process Eng..

[16] Cao Y., Li H., Sun J., Zhou X., Yao K., Nirere A. (2020). Nondestructive determination of the total mold colony count in green tea by hyperspectral imaging technology. J. Food Process Eng..

[17] Khulal U., Zhao J., Hu W., Chen Q. (2016). Nondestructive quantifying total volatile basic nitrogen (TVB-N) content in chicken using hyperspectral imaging (HSI) technique combined with different data dimension reduction algorithms. Food Chem..

[18] Sun J., Tang K., Wu X., Dai C., Chen Y., Shen J. (2018). Nondestructive identification of green tea varieties based on hyperspectral imaging technology. J. Food Process Eng..

[19] Kutsanedzie F., Guo Z., Chen Q. (2019). Advances in Nondestructive Methods for Meat Quality and Safety Monitoring. Food Rev. Int..

[20] Elmasry G., Kamruzzaman M., Sun D.-W., Allen P. (2012). Principles and Applications of Hyperspectral Imaging in Quality Evaluation of Agro-Food Products: A Review. Crit. Rev. Food Sci. Nutr..

[21] Min D., Zhao J., Bodner G., Ali M., Li F., Zhang X., Rewald B. (2023). Early decay detection in fruit by hyperspectral imaging–Principles and application Potential. Food Control.

[22] Lim H.T., Murukeshan V.M. (2016). Spatial-scanning hyperspectral imaging probe for bio-imaging applications. Rev. Sci. Instrum..

[23] Hartke J., Hagan N., Kinder B.A., Dereniak E.L. (2008). Computed tomographic imaging spectrometer (CTIS) and a snapshot hyperspectral imager and polarimeter. Int. J. High Speed Electron. Syst..

[24] Levin I.W., Bhargava R. (2005). Fourier transform infrared vibrational spectroscopic imaging: Integrating microscopy and molecular recognition. Annu. Rev. Phys. Chem..

[25] Wang P., Li J., Qi C., Wang L., Wang F.P. (2024). A method of reconstructing compressive spectral imaging with a complementary prior constraint. Opt. Commun..

[26] Sun J., Wu M., Hang Y., Lu B., Wu X., Chen Q. (2019). Estimating cadmium content in lettuce leaves based on deep brief network and hyperspectral imaging technology. J. Food Process Eng..

[27] Xu L., Chen Y., Feng A., Shi X., Feng Y., Yang Y., Wang Y., Wu Z., Zou Z., Ma W. (2023). Study on detection method of microplastics in farmland soil based on hyperspectral imaging technology. Environ. Res..

[28] Sun J., Lu X., Mao H., Wu X., Gao H. (2017). Quantitative Determination of Rice Moisture Based on Hyperspectral Imaging Technology and BCC-LS-SVR Algorithm. J. Food Process Eng..

[29] Zhou X., Sun J., Mao H., Wu X., Zhang X., Yang N. (2018). Visualization research of moisture content in leaf lettuce leaves based on WT-PLSR and hyperspectral imaging technology. J. Food Process Eng..

[30] Jiang S., Sun J., Xin Z., Mao H., Wu X., Li Q. (2017). Visualizing distribution of pesticide residues in mulberry leaves using NIR hyperspectral imaging. J. Food Process Eng..

[31] Chen L., Wu Y., Yang N., Sun Z. (2025). Advances in Hyperspectral and Diffraction Imaging for Agricultural Applications. Agriculture.

[32] Tian X.-Y., Aheto J.H., Dai C., Ren Y., Bai J.-W. (2021). Monitoring microstructural changes and moisture distribution of dry-cured pork. J. Sci. Food Agric..

[33] Tian X.-Y., Aheto J.H., Huang X., Zheng K., Dai C., Wang C., Bai J.-W. (2021). An evaluation of biochemical, structural and volatile changes of dry-cured pork. J. Sci. Food Agric..

[34] Xu Q., Bai J., Ma L., Li Z., Tan B., Sun L., Cai J. (2023). Identification of multiple symptoms of huanglongbing by electronic nose based on the variability of volatile organic compounds. Ann. Appl. Biol..

[35] Zubler A.V., Yoon J.-Y. (2020). Proximal Methods for Plant Stress Detection Using Optical Sensors and Machine Learning. Biosensors.

[36] Løkke M.M., Seefeldt H.F., Skov T., Edelenbos M. (2013). Color and textural quality of packaged wild rocket measured by multispectral Imaging. Postharvest Biol. Technol..

[37] Ouyang Q., Yang Y., Park B., Kang R., Wu J., Chen Q., Guo Z., Li H. (2020). A novel hyperspectral microscope imaging technology for rapid evaluation of particle size distribution in matcha. J. Food Eng..

[38] Cheng J., Sun J., Shi L., Dai C. (2024). An effective method fusing electronic nose and fluorescence hyperspectral imaging for the detection of pork freshness. Food Biosci..

[39] Cheng J., Sun J., Yao K., Xu M., Dai C. (2023). Multi-task convolutional neural network for simultaneous monitoring of lipid and protein oxidative damage in frozen-thawed pork using hyperspectral imaging. Meat Sci..

[40] Zhang Z., Zhu L. (2023). A review on unmanned aerial vehicle remote sensing: Platforms, sensors, data processing methods, and applications. Drones.

[41] Lopez-Ruiz N., Granados-Ortega F., Carvajal M.A., Martinez-Olmos A. (2017). Portable multispectral imaging system based on Raspberry Pi. Sens. Rev..

[42] Bian L., Wang Z., Zhang Y., Li L., Zhang Y., Yang C., Fang W., Zhao J., Zhu C., Meng Q. (2024). A broadband hyperspectral image sensor with high spatio-temporal resolution. Nature.

[43] Foster D.H., Amano K. (2019). Hyperspectral imaging in color vision research: Tutorial. J. Opt. Soc. Am. A.

[44] Qian S.E. (2021). Hyperspectral Satellites, Evolution, and Development History. IEEE J. Sel. Top. Appl. Earth Obs. Remote Sens..

[45] Seidel F.C., Stavros E.N., Cable M.L., Green R., Freeman A. (2018). Imaging spectrometer emulates Landsat: A case study with Airborne Visible Infrared Imaging Spectrometer (AVIRIS) and Operational Land Imager (OLI) data. Remote Sens. Environ..

[46] Cogliati S., Sarti F., Chiarantini L., Cosi M., Lorusso R., Lopinto E., Miglietta F., Genesio L., Guanter L., Damm A. (2021). The PRISMA imaging spectroscopy mission: Overview and first performance analysis. Remote Sens. Environ..

[47] Storch T., Honold H.-P., Chabrillat S., Habermeyer M., Tucker P., Brell M., Ohndorf A., Wirth K., Betz M., Kuchler M. (2023). The EnMAP imaging spectroscopy mission towards operations. Remote Sens. Environ..

[48] Thompson D.R., Green R.O., Bradley C., Brodrick P.G., Mahowald N., Ben Dor E., Bennett M., Bernas M., Carmon N., Chadwick K.D. (2024). On-orbit calibration and performance of the EMIT imaging spectrometer. Remote Sens. Environ..

[49] Cheng S., Yang X., Yang G., Chen B., Chen D., Wang J., Ren K., Sun W. (2024). Using ZY1-02D satellite hyperspectral remote sensing to monitor landscape diversity and its spatial scaling change in the Yellow River Estuary. Int. J. Appl. Earth Obs. Geoinf..

[50] Liu Y.-N., Sun D.-X., Hu X.-N., Liu S.-F., Cao K.-Q. (2020). AHSI: The Hyperspectral Imager on China’s GaoFen-5 Satellite. IOP Conf. Ser. Earth Environ. Sci..

[51] Liu H., Bruning B., Garnett T., Berger B. (2020). Hyperspectral imaging and 3D technologies for plant phenotyping: From satellite to close-range sensing. Comput. Electron. Agric..

[52] Özdogan G., Lin X., Sun D.W. (2021). Rapid and noninvasive sensory analyses of food products by hyperspectral imaging: Recent application developments. Trends Food Sci. Technol..

[53] Guo H., Yang K., Wu F., Chen Y., Shen J. (2023). Regional Inversion of Soil Heavy Metal Cr Content in Agricultural Land Using Zhuhai-1 Hyperspectral Images. Sensors.

[54] Bai J., Zhu S., Hao Y., Li X., Yang C., Wang C., Qiao X., Feng M., Xiao L., Song X. (2025). Comparative analysis of the effects of different dimensionality reduction algorithms on hyperspectral estimation of total nitrogen content in wheat soils. Eur. J. Agron..

[55] Tian Y., Sun J., Zhou X., Wu X., Lu B., Dai C. (2020). Research on apple origin classification based on variable iterative space shrinkage approach with stepwise regression-support vector machine algorithm and visible-near infrared hyperspectral imaging. J. Food Process Eng..

[56] Wang A., Dong S., Wang H., Iwahori Y. (2024). Multimodal semantic collaborative classification for hyperspectral images and LiDAR data. Remote Sens..

[57] Flynn K.C., Baath G., Lee T.O., Gowda P., Northup B. (2023). Hyperspectral reflectance and machine learning to monitor legume biomass and nitrogen accumulation. Comput. Electron. Agric..

[58] Dung C.D., Trueman S.J., Wallace H.M., Farrar M.B., Gama T., Tahmasbian I., Bai S.H. (2023). Hyperspectral imaging for estimating leaf, flower, and fruit macronutrient concentrations and predicting strawberry yields. Environ. Sci. Pollut. Res..

[59] Zhang H., Zhao J., Huang L., Huang W., Dong Y., Ma H., Ruan C. (2024). Development of new indices and use of CARS-Ridge algorithm for wheat fusarium head blight detection using in-situ hyperspectral data. Biosyst. Eng..

[60] Chen Y., Wang X., Zhang X., Sun Y., Sun H., Wang D., Xu X. (2023). Spectral Quantitative Analysis and Research of Fusarium Head Blight Infection Degree in Wheat Canopy Visible Areas. Agronomy.

[61] Zhou X., Sun J., Tian Y., Chen Q., Wu X., Hang Y. (2020). A deep learning based regression method on hyperspectral data for rapid prediction of cadmium residue in lettuce leaves. Chemom. Intell. Lab. Syst..

[62] Zhang L., An D., Wei Y., Liu J., Wu J. (2022). Prediction of oil content in single maize kernel based on hyperspectral imaging and attention convolution neural network. Food Chem..

[63] Jadesha G., Castelino E., Mahadevu P., Kitturmath M.S., Lohithaswa H.C., Karjagi C.G., Deepak D. (2025). Smart solutions for maize farmers: Machine learning-enabled web applications for downy mildew management and enhanced crop yield in India. Eur. J. Agron..

[64] Gao S., Xu J.H. (2024). Non-destructive detection of total acid of red globe grapes based on map fusion technique. Smart Agric. Technol..

[65] Yang C., Xu J., Feng M., Bai J., Sun H., Song L., Wang C., Yang W., Xiao L., Zhang M. (2023). Evaluation of Hyperspectral Monitoring Model for Aboveground Dry Biomass of Winter Wheat by Using Multiple Factors. Agronomy.

[66] Zhang L., Wang A., Zhang H., Zhu Q., Zhang H., Sun W., Niu Y. (2024). Estimating Leaf Chlorophyll Content of Winter Wheat from UAV Multispectral Images Using Machine Learning Algorithms under Different Species, Growth Stages, and Nitrogen Stress Conditions. Agriculture.

[67] Hu C., Zhao T., Duan Y., Zhang Y., Wang X., Li J., Zhang G. (2025). Visible-near infrared hyperspectral imaging for non-destructive estimation of leaf nitrogen content under water-saving irrigation in protected tomato cultivation. Front. Plant Sci..

[68] Zhang L., Wang X., Zhang H., Zhang B., Zhang J., Hu X., Du X., Cai J., Jia W., Wu C. (2024). UAV-Based Multispectral Winter Wheat Growth Monitoring with Adaptive Weight Allocation. Agriculture.

[69] Wei L., Yang H., Niu Y., Zhang Y., Xu L., Chai X. (2023). Wheat biomass, yield, and straw-grain ratio estimation from multi-temporal UAV-based RGB and multispectral images. Biosyst. Eng..

[70] Clevers J.G.P.W., Gitelson A.A. (2013). Remote estimation of crop and grass chlorophyll and nitrogen content using red-edge bands on Sentinel-2 and-3. Int. J. Appl. Earth Obs. Geoinf..

[71] Vincini M., Frazzi E. (2011). Comparing narrow and broad-band vegetation indices to estimate leaf chlorophyll content in planophile crop canopies. Precis. Agric..

[72] Mutanga O., Skidmore A.K. (2007). Red edge shift and biochemical content in grass canopies. ISPRS J. Photogramm. Remote Sens..

[73] Zhang J., Dai L., Huang Z., Gong C., Chen J., Xie J., Qu M. (2025). Corn Seed Quality Detection Based on Spectroscopy and Its Imaging Technology: A Review. Agriculture.

[74] Fu L., Sun J., Wang S., Xu M., Yao K., Cao Y., Tang N. (2022). Identification of maize seed varieties based on stacked sparse autoencoder and near-infrared hyperspectral imaging technology. J. Food Process. Eng..

[75] Yang H., Ming B., Nie C., Xue B., Xin J., Lu X., Xue J., Hou P., Xie R., Wang K. (2022). Maize Canopy and Leaf Chlorophyll Content Assessment from Leaf Spectral Reflectance: Estimation and Uncertainty Analysis across Growth Stages and Vertical Distribution. Remote Sens..

[76] Shi J., Wang Y., Li Z., Huang X., Shen T., Zou X. (2021). Simultaneous and nondestructive diagnostics of nitrogen/magnesium/potassium-deficient cucumber leaf based on chlorophyll density distribution features. Biosyst. Eng..

[77] Tang Z., Cai Y., Xiang Y., Lu J., Sun T., Shi H., Liu X., Zhang X., Li Z., Zhang F. (2025). Nitrogen nutritional diagnosis of summer maize (*Zea mays* L.) based on a hyperspectral data collaborative approach-evaluation of the estimation potential of three-dimensional spectral indices. Comput. Electron. Agric..

[78] Guo J., Zhang J., Xiong S., Zhang Z., Wei Q., Zhang W., Feng W., Ma X. (2021). Hyperspectral assessment of leaf nitrogen accumulation for winter wheat using different regression modeling. Precis. Agric..

[79] Oliveira R.A., Näsi R., Korhonen P., Mustonen A., Niemeläinen O., Koivumäki N., Hakala T., Suomalainen J., Kaivosoja J., Honkavaara E. (2024). High-precision estimation of grass quality and quantity using UAS-based VNIR and SWIR hyperspectral cameras and machine learning. Precis. Agric..

[80] Peng Y., Zhong W., Peng Z., Tu Y., Xu Y., Li Z., Liang J., Huang J., Liu X., Fu Y. (2024). Enhanced estimation of rice leaf nitrogen content via the integration of hybrid preferred features and deep learning methodologies. Agronomy.

[81] Tang Z., Wang X., Xiang Y., Liang J., Guo J., Li W., Lu J., Du R., Li Z., Zhang F. (2024). Application of hyperspectral technology for leaf function monitoring and nitrogen nutrient diagnosis in soybean (*Glycine max* L.) production systems on the Loess Plateau of China. Eur. J. Agron..

[82] Zhou H., Huang F., Lou W., Gu Q., Ye Z., Hu H., Zhang X. (2025). Yield prediction through UAV-based multispectral imaging and deep learning in rice breeding trials. Agric. Syst..

[83] Yu S., Fan J., Lu X., Wen W., Shao S., Guo X., Zhao C. (2022). Hyperspectral technique combined with deep learning algorithm for prediction of phenotyping traits in lettuce. Front. Plant Sci..

[84] Zhong Y., Sun J., Yao K., Cheng J., Du X. (2024). Detection of rice (with husk) moisture content based on hyperspectral imaging technology combined with MSLPP-ESMA-SVR model. J. Food Saf..

[85] Yan H., Zhang C., Gerrits M.C., Acquah S.J., Zhang H., Wu H., Zhao B., Huang S., Fu H. (2018). Parametrization of aerodynamic and canopy resistances for modeling evapotranspiration of greenhouse cucumber. Agric. For. Meteorol..

[86] Yan H.F., Zhang C., Hiroki O. (2018). Parameterization of canopy resistance for modeling the energy partitioning of a paddy rice field. Paddy Water Environ..

[87] Yan H., Acquah S.J., Zhang C., Wang G., Huang S., Zhang H., Zhao B., Wu H. (2019). Energy partitioning of greenhouse cucumber based on the application of Penman-Monteith and Bulk Transfer models. Agric. Water Manag..

[88] Abubaker A.B., Yan H., Li H., Wu Y., Elshaikh N.A., Hussein G., Pandab S., Hassan S. (2019). Enhancement of Depleted Loam Soil as Well as Cucumber Productivity Utilizing Biochar Under Water Stress. Commun. Soil Sci. Plant Anal..

[89] Javed Q., Wu Y., Xing D., Azeem A., Ullah I., Zaman M. (2017). Re-watering: An effective measure to recover growth and photosynthetic characteristics in salt-stressed *Brassica napus* L.. Chil. J. Agric. Res..

[90] Zhang C., Zhang W., Yan H., Ni Y., Akhlaq M., Zhou J., Xue R. (2022). Effect of micro–spray on plant growth and chlorophyll fluorescence parameter of tomato under high temperature condition in a greenhouse. Sci. Hortic..

[91] Xue R., Zhang C., Yan H., Li J., Ren J., Akhlaq M., Hameed M.U., Disasa K.N. (2023). Physiological Response of Tomato and Cucumber Plants to Micro-Spray in High-Temperature Environment. Agronomy.

[92] Ji L., Zhang Y., Alonso L., Li J., Qian Z., Li Z. (2018). Seasonal variations of sun-induced chlorophyll fluorescence from leaf to canopy level and its relations with plant functional traits for paddy-rice. American Geophysical Union, Fall Meeting 2018.

[93] Kimm H., Guan K., Burroughs C.H., Peng B., Ainsworth E.A., Bernacchi C.J., Moore C.E., Kumagai E., Yang X., Berry J.A. (2021). Quantifying high-temperature stress on soybean canopy photosynthesis: The unique role of sun-induced chlorophyll fluorescence. Glob. Change Biol..

[94] Cogato A., Pagay V., Marinello F., Meggio F., Grace P., De Antoni Migliorati M. (2019). Assessing the Feasibility of Using Sentinel-2 Imagery to Quantify the Impact of Heatwaves on Irrigated Vineyards. Remote Sens..

[95] Zhou X., Sun J., Zhang Y., Tian Y., Yao K., Xu M. (2021). Visualization of heavy metal cadmium in lettuce leaves based on wavelet support vector machine regression model and visible-near infrared hyperspectral imaging. J. Food Process. Eng..

[96] Zhang Y., Sun J., Li J., Wu X., Dai C. (2018). Quantitative Analysis of Cadmium Content in Tomato Leaves Based on Hyperspectral Image and Feature Selection. Appl. Eng. Agric..

[97] Shen G., Cao Y., Yin X., Dong F., Xu J., Shi J., Lee Y.-W. (2022). Rapid and nondestructive quantification of deoxynivalenol in individual wheat kernels using near-infrared hyperspectral imaging and chemometrics. Food Control.

[98] Zhang D., Wang Q., Lin F., Yin X., Gu C., Qiao H. (2020). Development and Evaluation of a New Spectral Disease Index to Detect Wheat Fusarium Head Blight Using Hyperspectral Imaging. Sensors.

[99] Huang L., Zhang H., Huang W., Dong Y., Ye H., Ma H., Zhao J. (2021). Identification of Fusarium head blight in wheat ears using vertical angle-based reflectance spectroscopy. Arab. J. Geosci..

[100] Qiu J., Gu H., Wang S., Ji F., He C., Jiang C., Shi J., Liu X., Shen G., Lee Y.-W. (2024). A diverse Fusarium community is responsible for contamination of rice with a variety of Fusarium toxins. Food Res. Int..

[101] Femenias A., Llorens-Serentill E., Ramos A.J., Sanchis V., Marín S. (2022). Near-infrared hyperspectral imaging evaluation of Fusarium damage and DON in single wheat kernels. Food Control.

[102] Chen X., Wu D., Wu X., Mokoena M., Olaniran A., Shi J., Xu J., Dong F. (2023). Effect of cleaning, milling, and cooking on the reduction of deoxynivalenol in freshly harvested rice. World Mycotoxin J..

[103] Meng Y.Q., Weng H.Y., Cen H.Y., Li H.Y., He Y. (2019). Carbohydrate metabolism and near-infrared spectral characteristics in asymptomatic Huang-longbing infected leaves. J. Agric. Food Chem..

[104] Qiu H., Liu T., Kong L., Yu X., Wang X., Huang M. (2024). Rapid Detection of Citrus Huanglongbing Based on Extraction of Characteristic Wavelength of Visible Spectrum and Classification Algorithm. Spectrosc. Spectr. Anal..

[105] Vieira J.G., Santana E.D., Conceição F.G., Filho F.H.I., de Pazini J.B., Rodrigues R., Yamamoto P.T. (2024). ‘*Candidatus Liberibacter asiaticus*’ infection alters the reflectance profile in asymptomatic citrus plants. Pest Manag. Sci..

[106] Deng X., Zhu Z., Yang J., Zheng Z., Huang Z., Yin X., Wei S., Lan Y. (2020). Detection of Citrus Huanglongbing Based on Multi-Input Neural Network Model of UAV Hyperspectral Remote Sensing. Remote Sens..

[107] Xu Q., Cai J.R., Zhang W., Bai J.W., Li Z.Q., Tan B., Sun L. (2022). Detection of citrus Huanglongbing (HLB) based on the HLB-induced leaf starch accumulation using a home-made computer vision system. Biosyst. Eng..

[108] Zhu H., Wang D., Wei Y., Zhang X., Li L. (2024). Combining Transfer Learning and Ensemble Algorithms for Improved Citrus Leaf Disease Classification. Agriculture.

[109] Chen T., Zeng R., Guo W., Hou X., Lan Y., Zhang L. (2018). Detection of Stress in Cotton (*Gossypium hirsutum* L.) Caused by Aphids Using Leaf Level Hyperspectral Measurements. Sensors.

[110] Luo J., Huang W., Yuan L., Zhao C., Du S., Zhang J., Zhao J. (2012). Evaluation of spectral indices and continuous wavelet analysis to quantify aphid infestation in wheat. Precis. Agric..

[111] Luo J., Huang W., Zhao J., Zhang J., Zhao C., Ma R. (2014). Detecting Aphid Density of Winter Wheat Leaf Using Hyperspectral Measurements. IEEE J. Sel. Top. Appl. Earth Obs. Remote Sens..

[112] Kumar J., Vashisth A., Sehgal V.K., Gupta V.K. (2012). Assessment of Aphid Infestation in Mustard by Hyperspectral Remote Sensing. J. Indian Soc. Remote Sens..

[113] Chen X., Hassan M., Yu J., Zhu A., Han Z., He P., Chen Q., Li H., Ouyang Q. (2024). Time series prediction of insect pests in tea gardens. J. Sci. Food Agric..

[114] Jang G., Choi N.J., Wen J., Kim H.-J., Chung Y.-S. (2024). The Trap of Presumption: A Case Study of Agricultural Research for Early Detection of Brown Planthopper Damage in Rice Fields. Korean J. Breed. Sci..

[115] Orlando V.S.W., Galo M.D.L.B.T., Martins G.D., Lingua A.M., Assis G.A., Belcore E. (2024). Hyperspectral Characterization of Coffee Leaf Miner (*Leucoptera coffeella*) (Lepidoptera: Lyonetiidae) Infestation Levels: A Detailed Analysis. Agronomy.

[116] Zhao G., Zhang Y., Lan Y., Deng J., Zhang Q., Zhang Z., Li Z., Liu L., Huang X., Ma J. (2023). Application Progress of UAV-LARS in Identification of Crop Diseases and Pests. Agronomy.

[117] Wan L., Li H., Wang A., Yang Y., Li C., Wang P. (2022). Hyperspectral Sensing of Plant Diseases: Principle and Methods. Agronomy.

[118] Ding S., Zhang X., Shang K., Xiao Q., Wang W., Rehman A.U. (2024). Removal of environmental influences for estimating soil texture fractions based on ZY1 satellite hyperspectral images. Catena.

[119] Wu Y.Q., Zhang B., Song K.S., Liu H.J., Wang Z.M., Liu D.W. (2011). Retrieval of soil organic matter content from hyper-spectra in Songnen Plain. J. Grad. Sch. Chin. Acad. Sci..

[120] Zhang F., Sheng K., Chen T., Hou J., Mei X. (2026). Effects of Precipitation Regimes on Ecosystem Respiration in Agricultural Regions of the Southern Tibetan Plateau. Agriculture.

[121] Bonifazi G., Fiore L., Gasbarrone R., Hennebert P., Serranti S. (2021). Detection of Brominated Plastics from E-Waste by Short-Wave Infrared Spectroscopy. Recycling.

[122] Ali M.A., Lyu X., Ersan M.S., Xiao F. (2024). Critical evaluation of hyperspectral imaging technology for detection and quantification of microplastics in soil. J. Hazard. Mater..

[123] Ai W., Chen G., Yue X., Wang J. (2023). Application of hyperspectral and deep learning in farmland soil microplastic detection. J. Hazard. Mater..

[124] Solangi K.A., Siyal A.A., Wu Y., Abbasi B., Solangi F., Lakhiar I.A., Zhou G. (2019). An Assessment of the Spatial and Temporal Distribution of Soil Salinity in Combination with Field and Satellite Data: A Case Study in Sujawal District. Agronomy.

[125] Deng M., Zhang C., Tang M., Liao C., Hu Y., Zhang Z., Feng S., Zheng Z. (2025). Stacking ensemble learning coupled with multi-source remote sensing data: Enhancing soil salinity inversion accuracy in barley-cultivated salinized soils. Agric. Water Manag..

[126] Cui X., Han W., Zhang H., Dong Y., Ma W., Zhai X., Zhang L., Li G. (2023). Estimating and mapping the dynamics of soil salinity under different crop types using Sentinel-2 satellite imagery. Geoderma.

[127] Zhang H., Wang Y., Liu L., Zhou J., Wan Q., Chen J., Cao Y., Zhang L., Feng F., Ning Q. (2024). Bibliometric Analysis of Contemporary Research on the Amelioration of Saline Soils. Agronomy.

[128] Chen M., Yu X., Su S., Lin D. Image Texture Analysis Based on Gray Level Co-Occurrence Matrix. Proceedings of the 2023 13th International Conference on Information Technology in Medicine and Education (ITME).

[129] Goldberg-Yehuda N., Nachshon U., Assouline S., Mau Y. (2024). The effect of mechanical compaction on the soil water retention curve: Insights from a rapid image analysis of micro-CT scanning. Catena.

[130] Kwasniewski A., Bak S., Ciężowski P., Knap L., Maciejewski J., Bialas M., Spagnuolo M. (2025). Visual tracking of plate compactor trajectories and soil compaction through metamaterial-inspired approach. Contin. Mech. Thermodyn..

[131] Arano V.E.R., Corinto L.M., dos Santos Reis J.M.P., Serafim M.E., Silva S.H.G., Barbosa S.M., Silva B.M. (2025). Proximal sensors fusion and machine learning algorithm combined to improve soil compaction prediction. Comput. Electron. Agric..

[132] Yang C., Guo Z., Barbin D.F., Dai Z., Watson N., Povey M., Zou X. (2025). Hyperspectral imaging and deep learning for quality and safety inspection of fruits and vegetables: A review. J. Agric. Food Chem..

[133] Xing F., Chen M. (2026). Advances in hyperspectral imaging for nondestructive food quality and safety detection. Foods.

[134] Tian Y., Sun J., Zhou X., Yao K., Tang N. (2022). Detection of soluble solid content in apples based on hyperspectral technology combined with deep learning algorithm. J. Food Process. Preserv..

[135] Fan S., Li J., Xia Y., Tian X., Guo Z., Huang W. (2019). Long-term evaluation of soluble solids content of apples with biological variability by using near-infrared spectroscopy and calibration transfer method. Postharvest Biol. Technol..

[136] Yuan L.-M., Cai J.-R., Sun L., Han E., Ernest T. (2016). Nondestructive Measurement of Soluble Solids Content in Apples by a Portable Fruit Analyzer. Food Anal. Methods.

[137] Guo C., Han C., Yan H., Li L. (2021). Nondestructive Analysis of Soluble Solids Content in Apple with a Portable NIR Spectrometer. Proceedings of the International Conference on NIR, Beijing, China, 18–21 October 2021.

[138] Xu M., Sun J., Yao K., Wu X., Shen J., Cao Y., Zhou X. (2022). Nondestructive detection of total soluble solids in grapes using VMD-RC and hyperspectral imaging. J. Food Sci..

[139] Xu M., Sun J., Cheng J., Yao K., Wu X., Zhou X. (2023). Non-destructive prediction of total soluble solids and titratable acidity in Kyoho grape using hyperspectral imaging and deep learning algorithm. Int. J. Food Sci. Technol..

[140] Ouyang Q., Wang L., Park B., Kang R., Chen Q. (2021). Simultaneous quantification of chemical constituents in matcha with visible-near infrared hyperspectral imaging technology. Food Chem..

[141] Zhu G., Zheng S., Xu Q., Qiu M., Wang H., Weng S. (2023). Detection of fungal infection in apple using hyperspectral transformation of RGB images with kernel regression. Postharvest Biol. Technol..

[142] Shi L., Sun J., Zhang B., Wu Z., Jia Y., Yao K., Zhou X. (2024). Simultaneous detection for storage condition and storage time of yellow peach using HSI with multi-target characteristic selection and multi-task model. J. Food Compos. Anal..

[143] Xing M., Long Y., Wang Q., Tian X., Fan S., Zhang C., Huang W. (2023). Physiological Alterations and Nondestructive Test Methods of Crop Seed Vigor: A Comprehensive Review. Agriculture.

[144] Sun J., Nirere A., Dusabe K.D., Zhong Y., Adrien G. (2024). Rapid and nondestructive watermelon (*Citrullus lanatus*) seed viability detection based on visible near-infrared hyperspectral imaging technology and machine learning algorithms. J. Food Sci..

[145] Qi G., Liu C., Han J., Zhou Y., Li Y., Wang Y. (2025). Variety Identification of Corn Seeds Based on Hyperspectral Imaging and Residual Mamba 1D CNN. Foods.

[146] Wang S., Sun J., Fu L., Xu M., Tang N., Cao Y., Yao K., Jing J. (2022). Identification of red jujube varieties based on hyperspectral imaging technology combined with CARS-IRIV and SSA-SVM. J. Food Process. Eng..

[147] Huang H., Liu Y., Feng C., Zhu S., Zhang S., Sun L., Liu C. (2024). Detection of mechanical damage in corn seeds using hyperspectral imaging and the ResNeSt_E deep learning network. Agriculture.

[148] Feng L., Zhu S., Liu F., He Y., Bao Y., Zhang C. (2019). Hyperspectral imaging for seed quality and safety inspection: A review. Plant Methods.

[149] Sun J., Lu X., Mao H., Jin X., Wu X. (2017). A Method for Rapid Identification of Rice Origin by Hyperspectral Imaging Technology. J. Food Process. Eng..

[150] Jeong S.W., Lyu J.I., Jeong H., Baek J., Moon J.-K., Lee C., Choi M.-G., Kim K.-H., Park Y.-I. (2024). SUnSeT: Spectral unmixing of hyperspectral images for phenotyping soybean seed Traits. Plant Cell Rep..

[151] Fan Y., An T., Yao X., Long Y., Wang Q., Wang Z., Tian X., Chen L., Huang W. (2025). An interpretable nondestructive detection model for maize seed viability: Based on grouped hyperspectral image fusion and key biochemical indicators. Comput. Electron. Agric..

[152] Zhang L., Wei Y., Liu J., An D., Wu J. (2024). Maize seed fraud detection based on hyperspectral imaging and one-class learning. Eng. Appl. Artif. Intell..

[153] Pang L., Xiao J., Ma J., Yan L. (2020). Hyperspectral imaging technology to detect the vigor of thermal-damaged *Quercus variabilis* seeds. J. For. Res..

[154] Yang G., Tian X., Fan Y., Xiang D., An T., Huang W., Long Y. (2024). Identification of Peanut Kernels Infected with Multiple *Aspergillus flavus* Fungi Using Line-Scan Raman Hyperspectral Imaging. Food Anal. Methods.

[155] He P., Wu Y., Wang J., Ren Y., Ahmad W., Liu R., Ouyang Q., Jiang H., Chen Q. (2020). Detection of mites *Tyrophagus putrescentiae* and *Cheyletus eruditus* in flour using hyperspectral imaging system coupled with chemometrics. J. Food Process. Eng..

[156] Pandey B.K., Atkinson J.A., Sturrock C.J. (2021). Non-invasive Imaging of Rice Roots in Non-compacted and Compacted Soil. Bio-Protoc..

[157] Xia Y., Li D., Wang Y., Xi Q., Jiao T., Wei J., Chen X., Chen Q., Chen Q. (2025). Rapid identification of cod authenticity based on hyperspectral imaging technology. Spectrochim. Acta Part A.

[158] Ding Y., Zeng R., Jiang H., Guan X., Jiang Q., Song Z. (2024). Classification of tea quality grades based on hyperspectral imaging spatial information and optimization models. J. Food Meas. Charact..

[159] Ouyang Q., Wang L., Park B., Kang R., Wang Z., Chen Q., Guo Z. (2020). Assessment of matcha sensory quality using hyperspectral microscope imaging technology. LWT-Food Sci. Technol..

[160] Zareef M., Chen Q., Ouyang Q., Arslan M., Hassan M., Ahmad W., Viswadevarayalu A., Wang P., Ancheng W. (2019). Rapid screening of phenolic compounds in congou black tea (*Camellia sinensis*) during in vitro fermentation process using portable spectral analytical system coupled chemometrics. J. Food Process. Preserv..

[161] Li L., Li M., Cui Q., Liu Y., Chen Y., Wang Y., Zhang Z., Chen Q., Ning J. (2021). Rapid monitoring of black tea fermentation quality based on a solution-phase sensor array combined with UV-visible spectroscopy. Food Chem..

[162] Zhang L., Sun J., Zhou X., Nirere A., Wu X., Dai R. (2020). Classification detection of saccharin jujube based on hyperspectral imaging technology. J. Food Process. Preserv..

[163] Nirere A., Sun J., Atindana V.A., Hussain A., Zhou X., Yao K. (2022). A comparative analysis of hybrid SVM and LS-SVM classification algorithms to identify dried wolfberry fruits quality. J. Food Process. Preserv..

[164] Tang N., Sun J., Xu M., Yao K., Cao Y., Liu D. (2022). Identification of fumigated and dyed Lycium barbarum by hyperspectral imaging technology. J. Food Process. Eng..

[165] Nirere A., Sun J., Kama R., Atindana V.A., Nikubwimana F.D., Dusabe K.D., Zhong Y. (2023). Nondestructive detection of adulterated wolfberry (*Lycium chinense*) fruits based on hyperspectral imaging technology. J. Food Process. Eng..

[166] Shen T.T., Zou X.B., Shi J.Y., Li Z.H., Huang X.W., Xu Y.W., Chen W. (2016). Determination Geographical Origin and Flavonoids Content of Goji Berry Using Near-Infrared Spectroscopy and Chemometrics. Food Anal. Methods.

[167] Li Y.H., Zou X.B., Shen T.T., Shi J.Y., Zhao J.W., Holmes M. (2017). Determination of Geographical Origin and Anthocyanin Content of Black Goji Berry (*Lycium ruthenicum* Murr.) Using Near-Infrared Spectroscopy and Chemometrics. Food Anal. Methods.

[168] Li H., Luo X., Haruna S.A., Zareef M., Chen Q., Ding Z., Yan Y. (2023). Au-Ag OHCs-based SERS sensor coupled with deep learning CNN algorithm to quantify thiram and pymetrozine in tea. Food Chem..

[169] Zhu C.Y., Jiang H., Chen Q.S. (2022). High Precisive Prediction of Aflatoxin B1 in Pressing Peanut Oil Using Raman Spectra Combined with Multivariate Data Analysis. Foods.

[170] Mei C., Zhu J., Zhu W., Wang H., Jiang H. (2025). Combining Colorimetric Sensor Technology with Swarm Intelligence Feature Optimization Algorithm to Realize High Precision Identification of Aflatoxin-B1 Content in Peanut Oil. Food Anal. Methods.

[171] Alves J.A.V., Steffens C.A., da Silva J.C., Pansera-Espindola B., do Amarante C.V.T., Moreira M.A. (2022). Quality of ‘San Andreas’ strawberries and control of gray mold with essential melaleuca oil. J. Food Process. Preserv..

[172] Bonah E., Huang X., Yi R., Aheto J.H., Osae R., Golly M. (2019). Electronic nose classification and differentiation of bacterial foodborne pathogens based on support vector machine optimized with particle swarm optimization algorithm. J. Food Process. Eng..

[173] Tahir H.E., Zou X., Xiao J., Mahunu G.K., Shi J., Xu J.-L., Sun D.-W. (2019). Recent progress in rapid analyses of vitamins, phenolic, and volatile compounds in foods using vibrational spectroscopy combined with chemometrics: A review. Food Anal. Methods.

[174] Li W., Wu Y., Du L., Shang X., Shi J. (2025). Hyperspectral Imaging for Foreign Matter Detection in Foods: Advances, Challenges, and Future Directions. Foods.

[175] Aheto J.H., Huang X., Tian X., Bonah E., Ren Y., Alenyorege E.A., Dai C. (2019). Investigation into crystal size effect on sodium chloride uptake and water activity of pork meat using hyperspectral imaging. J. Food Process. Preserv..

[176] Cheng J., Sun J., Xu M., Zhou X. (2023). Nondestructive detection of lipid oxidation in frozen pork using hyperspectral imaging technology. J. Food Compos. Anal..

[177] Cheng J., Sun J., Yao K., Xu M., Zhou X. (2022). Nondestructive detection and visualization of protein oxidation degree of frozen-thawed pork using fluorescence hyperspectral imaging. Meat Sci..

[178] Cheng J., Sun J., Yao K., Dai C. (2023). Generalized and hetero two-dimensional correlation analysis of hyperspectral imaging combined with three-dimensional convolutional neural network for evaluating lipid oxidation in pork. Food Control.

[179] Sun J., Cheng J., Xu M., Yao K. (2024). A method for freshness detection of pork using two-dimensional correlation spectroscopy images combined with dual-branch deep learning. J. Food Compos. Anal..

[180] Sun J., Yang F., Cheng J., Wang S., Fu L. (2024). Nondestructive identification of soybean protein in minced chicken meat based on hyperspectral imaging and VGG16-SVM. J. Food Compos. Anal..

[181] Aheto J.H., Huang X., Tian X., Ren Y., Bonah E., Alenyorege E.A., Lv R., Dai C. (2019). Combination of spectra and image information of HSI data for fast prediction of lipid oxidation attributes in pork meat. J. Food Process. Eng..

[182] Yao K., Sun J., Chen C., Xu M., Zhou X., Cao Y., Tian Y. (2022). Non-destructive detection of egg qualities based on hyperspectral imaging. J. Food Eng..

[183] Yao K., Sun J., Cheng J., Xu M., Chen C., Zhou X., Dai C. (2022). Development of Simplified Models for Non-Destructive Hyperspectral Imaging Monitoring of S-ovalbumin Content in Eggs during Storage. Foods.

[184] Donovan J.W., Mapes C.J. (1976). A differential scanning calorimetric study of conversion of ovalbumin to S-ovalbumin in eggs. J. Sci. Food Agric..

[185] Zhang D., Chen X., Lin Z., Lu M., Yang W., Sun X., Battino M., Shi J., Huang X., Shi B. (2025). Nondestructive detection of pungent and numbing compounds in spicy hotpot seasoning with HSI and machine learning. Food Chem..

[186] Liang J., Wang Y., Shi Y., Huang X., Li Z., Zhang X., Zou X., Shi J. (2023). Non-destructive discrimination of homochromatic foreign materials in cut tobacco based on VIS-NIR hyperspectral imaging. J. Sci. Food Agric..

[187] Tian X., Fang Q., Zhang X., Yu S., Dai C., Huang X. (2024). Visualization of Moisture Content, Reducing Sugars, and Chewiness in Bread During Oral Processing Based on Hyperspectral Imaging Technology. Foods.

[188] Monjur O., Ahmed M., Chowdhary G., Kamruzzaman M. (2025). Agro-HSR: The first large-scale agricultural-focused hyperspectral dataset for deep learning-based image reconstruction and quality prediction. Comput. Electron. Agric..

[189] Zhang H., Feng S., Wu D., Zhao C., Liu X., Zhou Y., Wang S., Deng H., Zheng S. (2024). Hyperspectral Image Classification on Large-Scale Agricultural Crops: The Heilongjiang Benchmark Dataset, Validation Procedure, and Baseline Results. Remote Sens..

[190] Ge Y., Song S., Yu S., Zhang X., Li X. (2024). Rice seed classification by hyperspectral imaging system: A real-world dataset and a credible algorithm. Comput. Electron. Agric..

[191] Bechar M.E.A., Saab N.A., Assainova O., El Hafyani H., Settouti N., Elbouz M. (2026). HSI-AgriFoodAnomaly, a hyperspectral dataset for foreign object detection in agri-food inspection. Sci. Data.

[192] Brunnengraeber E., Cassidy T., Woodbridge D., Jani A.D., Sharma A.K. (2026). Handheld hyperspectral imaging dataset of annual sowthistle and little mallow under abiotic stress for machine learning. Data Brief..

[193] Zhong C., Zhang J., Wu S., Zhang Y. (2020). Cross-Scene Deep Transfer Learning with Spectral Feature Adaptation for Hyperspectral Image Classification. IEEE J. Sel. Top. Appl. Earth Obs. Remote Sens..

[194] De Souza R.R., Fernandes D.D.D.S., Diniz P.H.G.D. (2021). Honey authentication in terms of its adulteration with sugar syrups using UV–Vis spectroscopy and one-class classifiers. Food Chem..

[195] Ciursă P., Pauliuc D., Dranca F., Ropciuc S., Oroian M. (2021). Detection of honey adulterated with agave, corn, inverted sugar, maple and rice syrups using FTIR analysis. Food Control.

[196] Tahir H.E., Zou X.B., Shen T.T., Shi J.Y., Mariod A.A. (2016). Near-Infrared (NIR) Spectroscopy for Rapid Measurement of Antioxidant Properties and Discrimination of Sudanese Honeys from Different Botanical Origin. Food Anal. Methods.

[197] Lobre C., Le Goff T., Journot T., Baier N., Serres E., Rubaldo L., Rochette F., Marmonier F., Grezes C., Cassillo C. (2025). A New Generation of MCT-Based p-on-n Technology for Focal-Plane Arrays Ranging from SWIR to VLWIR. J. Electron. Mater..

[198] Du J., Zhao X., Su J., Li B., Duan X., Dong T., Lin H., Ren Y., Miao Y., Radamson H.H. (2025). Review of Short-Wavelength Infrared Flip-Chip Bump Bonding Process Technology. Sensors.

[199] Balasekaran S., Inada H., Iguchi Y., Katsuyama T., Sasaki T. (2018). Photocapacitance characterization of deep levels in InGaAs/GaAsSb type-II MQW photodiodes. Infrared Phys. Technol..

[200] Kwon J., Kim S., Lee J., Park C., Kim O., Xu B., Bae J., Kang S. (2019). Uncooled Short-Wave Infrared Sensor Based on PbS Quantum Dots Using ZnO NPs. Nanomaterials.

[201] MacDougal M.H., Mak C., Meitzner J., Strand T., Hood A.D., Geske J.C., Bessonov A., Medina-Salazar I., Lutfullin M. (2024). Small pixel SWIR imagers using InGaAs and CQDs. Infrared Technol. Appl. L. SPIE.

[202] Arroyo-Mora J.P., Kalacska M., Lucanus O., Laliberté R., Chen Y., Gorman J., Marion A., Coulas L., Barber H., Borshchova I. (2023). Development of a Novel Implementation of a Remotely Piloted Aircraft System over 25 kg for Hyperspectral Payloads. Drones.

[203] Xue Q., Gao X., Lu F., Ma J., Song J., Xu J. (2024). Development and Application of Unmanned Aerial High-Resolution Convex Grating Dispersion Hyperspectral Imager. Sensors.

[204] Zhu F., Qiao X., Zhang Y., Jiang J. (2024). Analysis and mitigation of illumination influences on canopy close-range hyperspectral imaging for the in situ detection of chlorophyll distribution of basil crops. Comput. Electron. Agric..

[205] Wang D.-M., Chen X., Guo H., Wang Q.-H., Sun L., Sun W.-J. (2024). Exploring the response mechanism of *Pseudomonas plecoglossicida* to high-temperature stress by transcriptomic analyses for 2-keto gluconic acid production. Food Biosci..

[206] Mushi G.E., Serugendo G.D.M., Burgi P.Y. (2023). Data management system for sustainable agriculture among smallholder farmers in Tanzania: Research-in-progress. Inf. Technol. Dev..

[207] Cimino A., Coniglio I.M., Corvello V., Longo F., Sagawa J.K., Solina V. (2024). Exploring small farmers behavioral intention to adopt digital platforms for sustainable and successful agricultural ecosystems. Technol. Forecast. Soc. Change.

[208] Osrof H.Y., Tan C.L., Angappa G., Yeo S.F., Tan K.H. (2023). Adoption of smart farming technologies in field operations: A systematic review and future research agenda. Technol. Soc..

[209] Xu X., Chen Y., Yin H., Wang X., Zhang X. (2024). Nondestructive detection of SSC in multiple pear (*Pyrus pyrifolia* Nakai) cultivars using Vis-NIR spectroscopy coupled with the Grad-CAM method. Food Chem..

[210] Eckstein B.A., Arlen R. (2021). IEEE PROJECT 4001–Standards for Characterization and Calibration of Hyperspectral Imaging Devices. Int. Arch. Photogramm. Remote Sens. Spat. Inf. Sci..

[211] Bartsch B.d.A., Rosin N.A., dos Santos U.J., Coblinski J.A., Pelegrino M.H.P., Rosas J.T.F., Poppiel R.R., Ortiz E.B., Kochinki V.C.V., Gallo P. (2024). A Step Forward in Hybrid Soil Laboratory Analysis: Merging Chemometric Corrections, Protocols and Data-Driven Methods. Remote Sens..

[212] Fu J., Liu J., Zhao R., Chen Z., Qiao Y., Li D. (2022). Maize disease detection based on spectral recovery from RGB images. Front. Plant Sci..

[213] Gudkov S.V., Matveeva T.A., Sarimov R.M., Simakin A.V., Stepanova E.V., Moskovskiy M.N., Dorokhov A.S., Izmailov A.Y. (2023). Optical Methods for the Detection of Plant Pathogens and Diseases (Review). AgriEngineering.

[214] Leenen M., Pätzold S., Tóth G., Welp G. (2022). A LUCAS-based mid-infrared soil spectral library: Its usefulness for soil survey and precision agriculture. J. Plant Nutr. Soil Sci..

[215] Zhang H., Su H., Shen T., Sun G., Wang Q. (2025). Integrating PROSPECT-D physics and adversarial domain adaptation resnet for robust cross-ecosystem plant traits estimation. Front. Plant Sci..

[216] Feng J., Zhang T., Zhang J., Shang R., Dong W., Shi G., Jiao L. (2025). S 4 DL: Shift-sensitive spatial–spectral disentangling learning for hyperspectral image unsupervised domain adaptation. IEEE Trans. Neural Netw. Learn. Syst..

[217] Biswal P., Avdijaj J., Parente A., Coussement A. (2024). Solving the Radiation Transfer Equation in Participating Media Using Physics Informed Neural Networks. Proceedings of the World Congress on Mechanical, Chemical, and Material Engineering, Barcelona, Spain, 22–24 August 2024.

[218] Hossain D.S.M., Gabor K., Pullanagari R.R., Ian J.Y., Alvaro O., James H. Multi-scale estimation of vegetation nitrogen concentration using a physically informed neural network. Proceedings of the 2024 International Conference on Machine Intelligence for GeoAnalytics and Remote Sensing (MIGARS).

[219] Zérah Y., Valero S., Inglada J. (2024). Physics-constrained deep learning for biophysical parameter retrieval from Sentinel-2 images: Inversion of the PROSAIL model. Remote Sens. Environ..

[220] Wang X., Cai H., Zheng Z., Yu L., Wang Z., Li L. (2020). Modelling root water uptake under deficit irrigation and rewetting in Northwest China. Agron. J..

[221] Zhang W., Cao H., Zhang W., Hanan J.S., Ge D., Cao J., Xia J.A., Xuan S., Liang W., Zhang L. (2020). An aboveground biomass partitioning coefficient model for rapeseed (*Brassica napus* L.). Field Crops Res..

[222] Li W., Zhang C., Ma T., Li W. (2021). Estimation of summer maize biomass based on a crop growth model. Emir. J. Food Agric..

[223] Okyere F.G.G., Mehrem S.L., Snoek B.L., Van den Ackerveken G., Abeln S. (2026). SST-MAE: Learning Spectral-Spatio-Temporal Representations from Plant Hyperspectral Time Series to Discover Complex Genotype-Phenotype Relations. bioRxiv.

[224] Popov V.S., Milenkovich T., Khakimov K.T., Koroleva T.V., Deomidov A.D., Davletshin R.V., Hamidullin K.A., Saptsova O.A., Yakovlev V.O., Shuklov I.A. (2025). SWIR and MWIR Room Temperature Sensitivity of Photoresistors Based on HgTe Colloidal Quantum Dots. J. Commun. Technol. Electron..

[225] Andrew P., Colli A., Malik S., So D., Bower C., Liu Y., Nenonen M., Tamminen J., Coenen F., Kallioinen S. (2025). 57-1: Invited Paper: Infrared Imaging and Sensing Using Colloidal Quantum-Dot Detectors. SID Symp. Dig. Tech. Pap..

[226] Yan Y., Liu H.-Y., Bian L., Dai Y.-Y., Zhang B.-N., Xue S.-M., Zhou Y., Xu J.-L., Wang S.-D. (2024). Origin and suppression of dark current for high-performance colloidal quantum dot short-wave infrared photodetectors. Mater. Horiz..

[227] Agrawal R., Kumar H., Lnu S.R. (2025). Efficient LLMs for Edge Devices: Pruning, Quantization, and Distillation Techniques. Proceedings of the 2025 International Conference on Machine Learning and Autonomous Systems (ICMLAS), Prawet, Thailand, 10–12 March 2025.

[228] Kaur V., Jindal M.K., Kumar M. (2026). Efficient and Lightweight Deep Learning Approaches for Hyperspectral Image Classification: A Comprehensive Review. Arch. Comput. Methods Eng..

[229] Lin Y., Li H., Li A., Shi Y., Zhuang S. (2024). Application of AI-driven cloud services in intelligent agriculture pest and disease prediction. Appl. Comput. Eng..

[230] Kim K., Lee K. (2021). An Implementation of Open Source-Based Software as a Service (SaaS) to Produce TOA and TOC Reflectance of High-Resolution KOMPSAT-3/3A Satellite Image. Remote Sens..

[231] Huan M., Li Y., Chi L., Zhan S. (2022). The Effects of Agricultural Socialized Services on Sustainable Agricultural Practice Adoption among Smallholder Farmers in China. Agronomy.

[232] Xu L., Yao J. (2022). Supply Chain Scheduling Optimization in an Agricultural Socialized Service Platform Based on the Coordination Degree. Sustainability.

